# SU(2)$^2$-invariant $G_2$-instantons

**DOI:** 10.1007/s00208-017-1636-x

**Published:** 2018-01-05

**Authors:** Jason D. Lotay, Goncalo Oliveira

**Affiliations:** 1 Department of Mathematics, University College London, London, WC1E 6BT UK; 2 Mathematics Department, Duke University, Durham, NC 27708-0320 USA

## Abstract

We initiate the systematic study of $G_2$-instantons with SU(2)$^2$-symmetry. As well as developing foundational theory, we give existence, non-existence and classification results for these instantons. We particularly focus on $\mathbb {R}^4\times S^3$ with its two explicitly known distinct holonomy $G_2$ metrics, which have different volume growths at infinity, exhibiting the different behaviour of instantons in these settings. We also give an explicit example of sequences of $G_2$-instantons where “bubbling” and “removable singularity” phenomena occur in the limit.

## Introduction

In this article we study $G_2$-instantons: these are examples of Yang–Mills connections on Riemannian manifolds whose holonomy group is contained in the exceptional Lie group $G_2$ (so-called $G_2$-manifolds). These connections are, in a sense, analogues of anti-self-dual connections in dimension 4, and are likewise hoped to be used to understand the geometry and topology of $G_2$-manifolds, via the construction of enumerative invariants. Our focus is on $G_2$-instantons on $G_2$-manifolds where both the connections and ambient $G_2$ geometry enjoy SU(2)$^2$-symmetry. In particular, as a $G_2$-manifold is Ricci flat, for it to admit continuous symmetries it must be noncompact. By restricting to this case, we are able to shed light on the still rather poorly understood theory of $G_2$-instantons, in an explicit setting. In particular, we give new existence and non-existence results for $G_2$-instantons. Furthermore, we can see how general theory works in practice, examine how the ambient geometry affects the $G_2$-instantons and give local models for the behaviour of $G_2$-instantons on compact $G_2$-manifolds.

### $G_2$-instantons

Let $(X^7,\varphi )$ be a $G_2$-manifold,^1^ which implies the 7-manifold $X^7$ is endowed with a 3-form $\varphi$ which is closed and determines a Riemannian metric *g* with respect to which $\varphi$ is also coclosed. We shall denote $*\varphi$ by $\psi$ for convenience. Let $P \rightarrow X$ be a principal bundle with structure group *G* which we suppose to be a compact and semisimple Lie group. A connection *A* on *P* is said to be a $G_2$-instanton if

1.1 $$\begin{aligned} F_A \wedge \psi = 0. \end{aligned}$$

Equivalently, $G_2$-instantons satisfy the following $G_2$-analogue of the “anti-self-dual” condition:

1.2 $$\begin{aligned} F_A\wedge \varphi =-*F_A. \end{aligned}$$

As far as the authors are aware, the first time $G_2$-instantons appeared in the literature was in [6]. This reference investigates generalizations of the anti-self-dual gauge equations, in dimension greater than 4, and $G_2$-instantons appear there as an example.

More recently, the study of $G_2$-instantons has gained a special interest, primarily due to Donaldson–Thomas’ suggestion [10] that it may be possible to use $G_2$-instantons to define invariants for $G_2$-manifolds, inspired by Donaldson’s pioneering work on anti-self-dual connections on 4-manifolds. Later Donaldson–Segal [9], Haydys [14], and Haydys–Walpuski [15] gave further insights regarding that possibility.

On a compact holonomy $G_2$-manifold $(X^7, \varphi )$ any harmonic 2-form is “anti-self-dual” as in (1.2), hence any complex line bundle *L* on *X* admits a $G_2$-instanton, namely that whose curvature is the harmonic representative of $c_1(L)$. However, the construction of non-abelian $G_2$-instantons on compact $G_2$-manifolds is much more involved. In the compact case, the first such examples were constructed by Walpuski [25], over Joyce’s $G_2$-manifolds (see [16]). Sá Earp and Walpuski’s work [22, 26] gives an abstract construction of $G_2$-instantons, and currently one example, on the other known class of compact $G_2$-manifolds, namely “twisted connected sums” (see [7, 17]).

The goal of this paper is to perform a general analysis of $G_2$-instantons on some noncompact $G_2$-manifolds. In the noncompact setting, the first examples of $G_2$-instantons where found by Clarke [8], and further examples were given by the second author in [20]. In this article we primarily study $G_2$-instantons on $\mathbb {R}^4 \times S^3$, which has two known complete and explicit $G_2$-holonomy metrics, namely: the Bryant–Salamon (BS) metric [5] and the Brandhuber et al. (BGGG) metric [2]. Both these metrics have $\{0\} \times S^3$ as an associative submanifold: such area-minimizing submanifolds in $G_2$-manifolds have both known and expected relationships with $G_2$-instantons, so studying these metrics allows us to verify known theory and test expectations. Of particular note is that the BS and BGGG metrics have different volume growths at infinity, and are in a sense analagous to the flat and Taub-NUT hyperkähler metrics on $\mathbb {R}^4$. Our results exhibit the similarities and differences in the existence theory for $G_2$-instantons for these metrics.

### Summary

The aim of the article is to start the systematic study of SU(2)$^2$-invariant $G_2$-instantons. We now summarize the organization of our paper and the main results.

Both the BS and BGGG metric have SU(2)$^2$ as a subgroup of their isometry group: in fact, SU(2)$^2$ acts with cohomogeneity-1. All known complete SU(2)$^2$-invariant $G_2$-manifolds of cohomogeneity-1 actually have SU(2)$^2\times U(1)$-symmetry. These facts are summarized in Sect. 2, where we also deduce the ODEs for SU(2)$^2$-invariant $G_2$-instantons. In Sect. 2.5, we give some explicit elementary solutions to the equations, namely flat connections and abelian ones. Already in this simple abelian setting we see a marked difference between the $G_2$-instantons for the BS and BGGG metric.

In Sect. 3 we focus on the BS metric, which has isometry group $\mathrm{SU}(2)^3$. This group also acts with cohomogeneity-1 and has a unique singular orbit which is the associative $S^3$. We describe $\mathrm{SU}(2)^3$-invariant $G_2$-instantons with gauge group $\mathrm{SU}(2)$. A dichotomy arises from the two possible homogeneous bundles over the associative $S^3$ on which the instantons can extend: let $P_1$ and $P_{{{\mathrm{id}}}}$ denote these two bundles.

In the $P_1$ case, by combining our study in Sect. 3 with our work in Sect. 4 we obtain our first main result.

#### Theorem 1

Let *A* be an irreducible $\mathrm{SU}(2)^2 \times U(1)$-invariant $G_2$-instanton with gauge group $\mathrm{SU}(2)$ on the BS metric. If *A* smoothly extends over $P_1$, then it is one of Clarke’s $G_2$-instantons in [8].

See Theorems 4 and 7 for more precise statements and an explicit formula for the instantons, and see Corollary 1 for a classification of the reducible instantons. Here we mention that Clarke’s $G_2$-instantons form a family $\lbrace A^{x_1} \rbrace$, parametrized by $x_1\ge 0$, and the curvature of these connections decays at infinity.

In the $P_{{{\mathrm{id}}}}$ case, we find (in Theorem 5) a new explicit $G_2$-instanton $A^{\lim }$. We show in Theorem 6 and Corollary 2 that $A^{\lim }$ is, in a certain (precise) sense, the limit of Clarke’s ones as $x_1 \rightarrow + \infty$. We state our second main result informally, which confirms expectations from [23, 24].

#### Theorem 2

Let $\lbrace A^{x_1} \rbrace$ be a sequence of Clarke’s $G_2$-instantons with $x_1 \rightarrow + \infty$.

- After a suitable rescaling, the family $\lbrace A^{x_1} \rbrace$ bubbles off an anti-self-dual connection transversely to the associative $S^3 = \lbrace 0 \rbrace \times S^3$.
- The connections $A^{x_1}$ converge uniformly with all derivatives to $A^{\lim }$ on every compact subset of $(\mathbb {R}^4 {\setminus } \{0\})\times S^3$.
- The functions $\vert F_{A^{x_1}} \vert ^2 - \vert F_{A_{\lim }} \vert ^2$ are integrable and converge to $8 \pi ^2 \delta _{\lbrace 0 \rbrace \times S^3}$, where $\delta _{\lbrace 0 \rbrace \times S^3}$ denotes the delta current associated with the associative $S^3$.

Whilst (a) gives the familiar “bubbling” behaviour of sequences of instantons, with curvature concentrating on an associative $S^3$ by (c), we can interpret (b) as a “removable singularity” phenomenon since $A^{\lim }$ is a smooth connection on $\mathbb {R}^4\times S^3$. In proving Theorem 2, we show that as $\lbrace A^{x_1} \rbrace$ bubbles along the associative $S^3$ one obtains a Fueter section, as in [9, 14, 27]. Here this is just a constant map from $S^3$ to the moduli space of anti-self dual connections on $\mathbb {R}^4$ (thought of as a fibre of the normal bundle), taking value at the basic instanton on $\mathbb {R}^4$. Since $8\pi ^2$ is the Yang–Mills energy of the basic instanton, we can also view (c) as the expected “conservation of energy”.

We also give a local existence result for $G_2$-instantons in a neighbourhood of the associative $S^3$ that extend over $P_{{{\mathrm{id}}}}$ in Proposition 4. The outcome is that there is a local one-parameter family of such instantons. Of these only one, i.e. $A^{\lim }$, is shown to extend over the whole of $\mathbb {R}^4 \times S^3$. The other ones may blow up at a finite distance to $\lbrace 0 \rbrace \times S^3$, as suggested by numeric simulations. Some of the necessary analysis leading to our local existence results is given in Appendix A.

In order to use similar techniques for $G_2$-intantons on the BGGG metric, we must reduce the symmetry group to $\mathrm{SU}(2)^2 \times U(1)$. This acts with cohomogeneity-1 both on BGGG and BS and, as before, its only singular orbit is the associative $\lbrace 0 \rbrace \times S^3$. Hence, in Sect. 4 we describe $\mathrm{SU}(2)^2 \times U(1)$-invariant $G_2$-instantons on cohomogeneity-1 metrics with that symmetry on $\mathbb {R}^4 \times S^3$. As a result, the same dichotomy appears in that the $G_2$-instantons can extend over the associative $S^3$ either on the homogeneous bundle $P_1$ or $P_{{{\mathrm{id}}}}$. We can thus compare the existence of $G_2$-instantons for the BS and BGGG metrics. While there is a 1-parameter family of $G_2$-instantons (Clarke’s ones) that smoothly extend over $P_1$ on the BS metric, for the BGGG metric we instead have the following.

#### Theorem 3

The moduli space of irreducible $\mathrm{SU}(2)^2 \times U(1)$-invariant $G_2$-instantons with gauge group $\mathrm{SU}(2)$ on the BGGG metric, smoothly extending on $P_1$, contains a nonempty (and unbounded) open set $U \subset \mathbb {R}^2$. Moreover, the following holds.

- The instantons in *U* have quadratically decaying curvature.
- The map $Hol_{\infty } : U \rightarrow U(1) \subset \mathrm{SU}(2)$, which evaluates the holonomy of the $G_2$-instanton along the finite size circle at $+\infty$, is surjective.

The more precise version of this result appears as Theorem 9 and Corollary 3. It is typical in gauge theory to assume a bound on the curvature of the connection. One might be tempted to impose an $L^2$-bound, but this is too restrictive in the $G_2$ setting: in particular, Clarke’s examples do not satisfy this. Therefore, we impose a weak natural curvature bound in deriving Theorem 3, namely that the curvature stays bounded. We also prove that there is a 2-parameter family of locally defined instantons on $P_1$ for the BGGG metric which do not extend globally with bounded curvature: this is Theorem 8.

Finally, we give local existence results for $G_2$-instantons with $\mathrm{SU}(2)^2 \times U(1)$-symmetry in a neighbourhood of an associative $S^3$, on any $\mathrm{SU}(2)^2 \times U(1)$-invariant $G_2$-metric. In Proposition 7, we show the existence of a 2-parameter family of locally defined $G_2$-instantons smoothly extending over $P_1$, whereas in Proposition 8 we show the existence of a 1-parameter family of $G_2$-instantons smoothly extending over $P_{{{\mathrm{id}}}}$. This yields the possibility for further study of $G_2$-instantons even on the well-known Bryant–Salamon metric on $\mathbb {R}^4\times S^3$.

## The SU(2)$^2$-invariant equations

In this section we derive the ordinary differential equations (ODEs) which describe invariant $G_2$-instantons on SU(2)$^2$-invariant $G_2$-manifolds of cohomogeneity-1. We begin by giving the general framework of the evolution equations approach to $G_2$-manifolds and $G_2$-instantons in Sect. 2.1. We then apply this theory in Sect. 2.2 to the case of the invariant $G_2$-manifolds we wish to study, leading to systems of ODEs describing the $G_2$-manifolds, and summarise the known complete examples which arise from this approach. We then give a short presentation of the theory of invariant fields on homogeneous bundles in Sect. 2.3 so that we can obtain the general expression for an invariant connection on a principal orbit and its curvature. Combining these considerations yields our desired ODEs in Sect. 2.4, which we then solve in elementary cases in Sect. 2.5.

### Evolution equations

In the work to be developed it is relevant to analyze the case when $X^7=I_t \times M^6$ and $I_t \subset \mathbb {R}$ is an interval with coordinate $t\in \mathbb {R}$. Let $(\omega (t), \Omega _2(t))$ be a 1-parameter family of $\mathrm{SU}(3)$-structures^2^ parametrized by $t \in I_t$ and write the $G_2$-structure

2.1 $$\begin{aligned} \varphi = dt \wedge \omega (t) + \Omega _1(t), \quad \psi = \frac{\omega ^2(t)}{2} - dt \wedge \Omega _2(t), \end{aligned}$$

where $\Omega _1(t)=J_t \Omega _2(t)$ and $J_t$ is the almost complex structure determined by $\Omega _2(t)$. This $G_2$-structure is torsion-free (i.e. $d\varphi =0$ and $d\psi =0$) if and only if the 1-parameter family $(\omega (t), \Omega _2(t))$ is a solution of the so-called “Hitchin flow”,^3^ i.e. if we write $\dot{f}=df/dt$, then

2.2 $$\begin{aligned} \dot{\Omega }_1= d\omega , \quad \omega \wedge \dot{\omega } = -d\Omega _2, \end{aligned}$$

subject to the constraints $d \Omega _1=0=d\omega ^2$ for all *t*, which means that $(\omega (t), \Omega _2(t))$ is a family of half-flat $\mathrm{SU}(3)$-structures solving (2.2). (In fact, it is enough to impose the half-flat condition on the $\mathrm{SU}(3)$-structure at some initial time and the evolution (2.2) will then preserve this condition.) For more on half-flat $\mathrm{SU}(3)$-structures, in a case relevant to us, the reader can see [19]. The resulting $G_2$-structure induces the metric $g=dt^2 + g_t$, where $g_t$ is the metric on $\lbrace t \rbrace \times M$ induced by the $\mathrm{SU}(3)$-structure $(\omega (t), \Omega _2(t))$.

In this situation our bundle *P* must be pulled back from *M* and, working in temporal gauge, $A=a(t)$ is a 1-parameter family of connections on *P*, so $F_A = dt \wedge \dot{a} + F_a(t)$. Hence *A* is a $G_2$-instanton, i.e. solves (1.1), if and only if

2.3 $$\begin{aligned} \dot{a} \wedge \frac{\omega ^2}{2} - F_a \wedge \Omega _2 =0, \quad F_a \wedge \frac{\omega ^2}{2} = 0. \end{aligned}$$

Using $*_t$ to denote the Hodge-$*$ associated with the $\mathrm{SU}(3)$ structure $(\omega (t), \Omega _2(t))$ we have

2.4 $$\begin{aligned} J_t \dot{a} = -*_t \left( \dot{a} \wedge \frac{\omega ^2}{2} \right) \quad \text {and}\quad \Lambda _t F_a = *_t \left( F_a \wedge \frac{\omega ^2}{2} \right) , \end{aligned}$$

where $\Lambda _t$ denotes the metric dual of the operation of wedging with $\omega$. Then, applying $*_t$ to both sides of (2.3) we have

2.5 $$\begin{aligned} J_t \dot{a}= & {} - *_t \left( F_a \wedge \Omega _2 \right) , \end{aligned}$$

2.6 $$\begin{aligned} \Lambda _t F_a= & {} 0. \end{aligned}$$

#### Lemma 1

Let $X=I_t\times M$ be equipped with a $G_2$-structure $\varphi$ as in (2.1) satisfying $\omega \wedge d \omega =0$ and $\omega \wedge \dot{\omega }=- d \Omega _2$, which is equivalent to $d\psi =0$. Then, $G_2$-instantons *A* for $\varphi$ are in one-to-one correspondence with 1-parameter families of connections $\lbrace a(t) \rbrace _{t \in I_t}$ solving the evolution equation

2.7 $$\begin{aligned} J_t \dot{a} = - *_t \left( F_a \wedge \Omega _2 \right) , \end{aligned}$$

subject to the constraint $\Lambda _t F_a =0$. Moreover, this constraint is compatible with the evolution: more precisely, if it holds for some $t_0 \in I_t$, then it holds for all $t \in I_t$.

#### Proof

The evolution equation and the constraint follow immediately from equations (2.5) and (2.6). To prove that the constraint is preserved by the evolution we compute

$$\begin{aligned} \frac{d}{dt} \left( F_a \wedge \omega ^2 \right)&= d_a \dot{a} \wedge \omega ^2 + F_a \wedge \frac{d}{dt}\omega ^2 = d_a (\dot{a} \wedge \omega ^2) -2 F_a \wedge d \Omega _2\\&= 2 d_a(F_a \wedge \Omega _2) - 2 F_a \wedge d \Omega _2 = 0, \end{aligned}$$

where we used (2.2), (2.3), (2.7) and the Bianchi identity $d_a F_a=0$. $\square$

#### Proposition 1

In the setting of Lemma 1, suppose that the family of $\mathrm{SU}(3)$-structures $(\omega (t), \Omega _2(t))$ depends real analytically on *t*, and let *a*(0) be a real analytic connection on *P* such that $\Lambda _0 F_a(0)=0$. Then there is $\epsilon >0$ and a $G_2$-instanton *A* on $(-\epsilon , \epsilon ) \times M^6$ with $A\vert _{\lbrace 0 \rbrace \times M^6}=a(0)$.

#### Proof

This is immediate from applying the Cauchy–Kovalevskaya theorem to (2.7). $\square$

### SU(2)$^2$-invariant $G_2$-manifolds of cohomogeneity-1

In this section we shall give a self-contained exposition of all the known complete SU(2)$^2$-invariant $G_2$-holonomy metrics. We shall see that all these examples actually have $\mathrm{SU}(2)^2 \times U(1)$-symmetry. We start with some preparation. Split the Lie algebra $\mathfrak {su}(2) \oplus \mathfrak {su}(2)$ as $\mathfrak {su}^+ \oplus \mathfrak {su}^-$, as follows. If $\lbrace T_i \rbrace _{i=1}^3$ is a basis for $\mathfrak {su}(2)$ such that $[T_i,T_j] = 2 \epsilon _{ijk} T_k$, then $T_i^+ = (T_i, T_i)$ and $T_i^-=(T_i, -T_i)$ for $i=1,2,3$ give a basis for $\mathfrak {su}^+$ and $\mathfrak {su}^-$ respectively. (Thus $\mathfrak {su}^+$ and $\mathfrak {su}^-$ are diagonal and anti-diagonal copies of $\mathfrak {su}(2)$ in $\mathfrak {su}(2)\oplus \mathfrak {su}(2)$.) We shall let $\lbrace \eta _i^+ \rbrace _{i=1}^{3}$ and $\lbrace \eta _i^- \rbrace _{i=1}^{3}$ be dual bases to $\{T_i^+\}_{i=1}^3$ and $\{T_i^-\}_{i=1}^3$ respectively. The Maurer–Cartan relations in this case give

2.8 $$\begin{aligned} d\eta _i^+= & {} -\epsilon _{ijk} \left( \eta _j^+ \wedge \eta _k^+ + \eta _j^- \wedge \eta _k^- \right) , \end{aligned}$$

2.9 $$\begin{aligned} d\eta _i^-= & {} -2\epsilon _{ijk} \eta _j^- \wedge \eta _k^+. \end{aligned}$$

The complement of the singular orbit can be written as $\mathbb {R}^+_t \times M$, where *M* denotes a principal orbit, which is a finite quotient of $S^3\times S^3$. The $\mathrm{SU}(2)\times \mathrm{SU}(2)$-invariant $\mathrm{SU}(3)$-structure on the principal orbit $\{t\}\times M$ is given by [19]

2.10 $$\begin{aligned} \omega= & {} 4 \sum _{i=1}^3 A_iB_i \eta _i^- \wedge \eta _i^+, \end{aligned}$$

2.11 $$\begin{aligned} \Omega _1= & {} 8 B_1B_2B_3 \eta _{123}^- - 4 \sum _{i,j,k} \epsilon _{ijk} A_i A_j B_k \eta _i^+ \wedge \eta _j^+ \wedge \eta _k^-, \end{aligned}$$

2.12 $$\begin{aligned} \Omega _2= & {} - 8 A_1A_2A_3 \eta _{123}^+ + 4 \sum _{i,j,k} \epsilon _{ijk} B_i B_j A_k \eta _i^- \wedge \eta _j^- \wedge \eta _k^+, \end{aligned}$$

for real-valued functions $A_i$, $B_i$ of $t \in \mathbb {R}^+$, where $\eta _{123}^{\pm }$ denotes $\eta _{1}^{\pm } \wedge \eta _{2}^{\pm } \wedge \eta _{3}^{\pm }$. For future reference, we remark that

$$\begin{aligned} 4 \sum _{i,j,k} \epsilon _{ijk} B_i B_j A_k \eta _i^- \wedge \eta _j^- \wedge \eta _k^+= 8 B_1 B_2 A_3 \eta _1^- \wedge \eta _2^- \wedge \eta _3^+ + \text {cyclic permutations}. \end{aligned}$$

The compatible metric determined by this $\mathrm{SU}(3)$ structure on $\{t\}\times M$ is [19]

2.13 $$\begin{aligned} g_t = \sum _{i=1}^3 (2A_i)^2 \eta _{i}^+ \otimes \eta _i^+ + (2B_i)^2 \eta _i^- \otimes \eta _i^-, \end{aligned}$$

and the resulting metric on $\mathbb {R}_t \times M$, compatible with the $G_2$-structure $\varphi = dt \wedge \omega + \Omega _1$, is given by $g=dt^2 + g_t$. Recall also that this metric has holonomy in $G_2$ if and only if the $\mathrm{SU}(3)$-structure above solves the Hitchin flow equations (2.2).

These considerations allow us to derive the general ODEs describing SU(2)$^2$-invariant $G_2$-manifolds of cohomogeneity-1 as follows (c.f. [19]):

2.14 $$\begin{aligned} \dot{A}_i= & {} \frac{1}{2}\left( \frac{A_i^2}{A_jA_k}-\frac{A_i^2}{B_jB_k} -\frac{A_j^2+A_k^2}{A_jA_k}+\frac{B_j^2+B_k^2}{B_jB_k}\right) , \end{aligned}$$

2.15 $$\begin{aligned} \dot{B}_i= & {} \frac{1}{2}\left( \frac{A_j^2+B_k^2}{A_jB_k} +\frac{A_k^2+B_j^2}{A_kB_j}-\frac{B_i^2}{A_jB_k}-\frac{B_i^2}{A_kB_j}\right) , \end{aligned}$$

where (*i*, *j*, *k*) denotes a cyclic permutation of (1, 2, 3). We will be interested in this article in the setting where we have known complete examples. In fact, in every such example there is an extra *U*(1)-symmetry: this *U*(1) acts diagonally on $S^3 \times S^3$ with infinitesimal generator $T_1^+$. As a consequence, we have $A_2=A_3$ and $B_2=B_3$ and (2.2) becomes (as in [1]):

2.16 $$\begin{aligned} \dot{A}_1&= \frac{1}{2}\left( \frac{A_1^2}{A_2^2}-\frac{A_1^2}{B_2^2}\right) , \end{aligned}$$

2.17 $$\begin{aligned} \dot{A}_2&= \frac{1}{2}\left( \frac{B_1^2+B_2^2-A_2^2}{B_1B_2} -\frac{A_1}{A_2}\right) ,\end{aligned}$$

2.18 $$\begin{aligned} \dot{B}_1&= \frac{A_2^2+B_2^2-B_1^2}{A_2B_2}, \end{aligned}$$

2.19 $$\begin{aligned} \dot{B}_2&= \frac{1}{2}\left( \frac{A_2^2+B_1^2-B_2^2}{A_2B_1} +\frac{A_1}{B_2}\right) . \end{aligned}$$

We now give the known examples of cohomogeneity-1 complete $G_2$-metrics with SU(2)$^2$-symmetry.

#### The Bryant–Salamon (BS) metric

The Bryant–Salamon metric on $\mathbb {R}^4\times S^3$ [5] is one of the first examples of a complete metric with $G_2$-holonomy. It is not only SU(2)$^2$-invariant, but actually $\mathrm{SU}(2)^3$-invariant, having group diagram $I(\mathrm{SU}(2)^3, \mathrm{SU}(2), \mathrm{SU}(2)^2)$; i.e. the principal orbits are $\mathrm{SU}(2)^3/\mathrm{SU}(2)\cong S^3\times S^3$ and the (unique) singular orbit is $\mathrm{SU}(2)^3/\mathrm{SU}(2)^2\cong S^3$. (Here, the $\mathrm{SU}(2)$ in $\mathrm{SU}(2)^3$ is the subgroup $\mathrm{SU}(2)_3 = 1 \times 1 \times \mathrm{SU}(2)$, and $\mathrm{SU}(2)^2 \subset \mathrm{SU}(2)^3$ is the subgroup $\mathrm{SU}(2)_3 \times \Delta \mathrm{SU}(2)$, where $\Delta \mathrm{SU}(2) \subset \mathrm{SU}(2)^2 \times 1$ is the diagonal.) In terms of the SU(2)$^2$-invariant point of view above, the metric can be explicitly written as follows.

In this case the extra symmetry means that $A_1=A_2=A_3$ and $B_1=B_2=B_3$ and the Eqs. (2.16)–(2.19) reduce to:

2.20 $$\begin{aligned} \dot{A}_1=\frac{1}{2}\left( 1-\frac{A_1^2}{B_1^2}\right) \quad \text {and}\quad \dot{B}_1=\frac{A_1}{B_1}. \end{aligned}$$

Setting $B_1=s$ and $A_1=sC(s)$ we see that (2.20) becomes $\frac{d}{ds}(sC)=\frac{1-C^2}{2C}$ which we can easily solve as $C(s)=\sqrt{\frac{1-c^3s^{-3}}{3}}$, so that, for $c>0$ and $s\ge c$,

2.21 $$\begin{aligned} A_1(s)=\frac{s}{\sqrt{3}}\sqrt{1-c^3s^{-3}}\quad \text {and}\quad B_1(s)=s. \end{aligned}$$

In particular, choosing $c=1$ and using *t*, the arc length parameter along the geodesic parametrized by *s*, we define a coordinate $r \in [1; + \infty )$ implicitly by

2.22 $$\begin{aligned} t(r)=\int _1^r \frac{ds}{\sqrt{1-s^{-3}}}, \end{aligned}$$

and solve (2.20) as follows:

2.23 $$\begin{aligned} A_1=A_2=A_3=\frac{r}{3}\sqrt{1-r^{-3}} \quad \text {and} \quad B_1=B_2=B_3=\frac{r}{\sqrt{3}}. \end{aligned}$$

It is easy to verify that the geometry at infinity is asymptotically conical to the standard holonomy $G_2$-cone on $S^3\times S^3$. In fact, we see from (2.21) that one obtains a one-parameter family^4^ of solutions to (2.20), equivalent up to scaling, whose limit with$c=0$ is the conical solution. Moreover, the torsion-free $G_2$-structure has a unique compact associative submanifold which is the singular orbit $\lbrace 0 \rbrace \times S^3\cong \mathrm{SU}(2)^2/\mathrm{SU}(2)$.

There is a one-parameter family of $\mathrm{SU}(2)^3$-invariant $G_2$-instantons for this Bryant–Salamon torsion-free $G_2$-structure constructed by Clarke [8], where the parameter can be interpreted as how concentrated the instanton is around the associative $S^3$. We shall prove, in Theorem 4 and Proposition 7, a uniqueness result for these $G_2$-instantons in the class of $\mathrm{SU}(2)^2 \times U(1)$-invariant ones.

##### Remark 1

In [5] Bryant–Salamon constructed $G_2$-holonomy metrics on the total spaces of the bundles of anti-self-dual 2-forms over $\mathbb {CP}^2$ and $\mathbb {S}^4$, i.e. $\Lambda ^2_- \mathbb {CP}^2$ and $\Lambda ^2_-\mathbb {S}^4$. Such metrics are also of cohomogeneity-1 with respect to SO(5) and SU(3) respectively and asymptotically conical. Instantons on these $G_2$-manifolds are also known to exist and some explicit examples can be found in [20].

It follows from Proposition 3 in [20] [or easily from (2.5), (2.6)] that on an asymptotically conical $G_2$-manifold, a $G_2$-instanton whose curvature is decaying pointwise at infinity will have as a limit (if it exists) a pseudo-Hermitian–Yang–Mills connection $a_{\infty }$ (or nearly Kähler instanton): i.e. if $\varphi _{\infty }=t^2dt\wedge \omega _{\infty }+t^3\Omega _{1,\infty }$ and $\psi _{\infty }=t^4\omega _{\infty }^2/2-t^3dt\wedge \Omega _{2,\infty }$ is the conical $G_2$-structure on the asymptotic cone then $F_{a_{\infty }}\wedge \omega _{\infty }^2=0$ and $F_{a_{\infty }}\wedge \Omega _{2,\infty }=0$.

#### The Brandhuber et al. (BGGG) metric

On $\mathbb {R}^4 \times S^3$ there is another complete $G_2$-holonomy metric constructed by Brandhuber and collaborators in [2], which is a member of a family of complete $\mathrm{SU}(2)^2\times U(1)$-invariant, cohomogeneity-1, $G_2$-holonomy metrics on $\mathbb {R}^4\times S^3$ found in [3].^5^ To derive this example one can choose $c>0$, set $B_1=s$ and

$$\begin{aligned} A_1=c\frac{ds}{dt}=c\frac{A_2^2+B_2^2-s^2}{A_2B_2} \end{aligned}$$

from (2.18). Letting $C_{\pm }=A_2^2\pm B_2^2$ the Eqs. (2.17) and (2.19) yield

$$\begin{aligned} \frac{d}{ds}C_+=\frac{s^2C_+-C_-^2}{s(C_+-s^2)}\quad \text {and}\quad \frac{d}{ds}C_-=\frac{C_-}{s}-2c. \end{aligned}$$

The second equation is easily integrated and so we are able to find solutions

$$\begin{aligned} C_+(s)=\frac{3s^2-c^2}{2}\quad \text {and}\quad C_-(s)=-cs. \end{aligned}$$

We thus obtain a one-parameter family of solutions to (2.16)–(2.19):

2.24 $$\begin{aligned} A_1(s)= & {} 2c\sqrt{\frac{s^2-c^2}{9s^2-c^2}},\quad A_2(s)=\frac{1}{2}\sqrt{(3s+c)(s-c)}, \end{aligned}$$

2.25 $$\begin{aligned} B_1(s)= & {} s,\quad B_2(s)=\frac{1}{2}\sqrt{(3s-c)(s+c)}, \end{aligned}$$

defined for $s\ge c>0$. These solutions give holonomy $G_2$ metrics on $\mathbb {R}^4\times S^3$ by Lemma 8 in Appendix A. We can further scale the metric from *g* to $\lambda ^2 g$ and the resulting fields scale as $A_i^{\lambda }(s)=\lambda A_i (s/\lambda )$, $B_i^{\lambda }(s)=\lambda B_i(s/\lambda )$. These give the following family of solution to the ODEs (2.16)–(2.19) above:

$$\begin{aligned} A_1^{\lambda }(s)= & {} 2c \lambda \sqrt{\frac{s^2-c^2\lambda ^2}{9s^2-c^2\lambda ^2}},\quad A_2^{\lambda }(s)=\frac{1}{2}\sqrt{(3s+c\lambda )(s-c \lambda )},\\ B_1^{\lambda }(s)= & {} s , \quad B_2^{\lambda }(s)=\frac{1}{2}\sqrt{(3s-c\lambda )(s+c\lambda )}. \end{aligned}$$

We see that under the scaling we have $c\mapsto c\lambda$, so we can always scale so that $c=1$. In particular, one can set $\lambda =3/2$, $c=1$ and as in [2] define the coordinate $r\in [9/4,+\infty )$ implicitly by

2.26 $$\begin{aligned} t(r)=\int _{9/4}^{r} \frac{\sqrt{(s-3/4)(s+3/4)}}{\sqrt{(s-9/4)(s+9/4)}} ds \end{aligned}$$

and find that

$$\begin{aligned} A_1= & {} \frac{\sqrt{(r-9/4)(r+9/4)}}{\sqrt{(r-3/4)(r+3/4)}}, \quad A_2=A_3=\sqrt{\frac{(r-9/4)(r+3/4)}{3}},\\ B_1= & {} \frac{2r}{3}, \quad B_2=B_3=\sqrt{\frac{(r-3/4)(r+9/4)}{3}} \end{aligned}$$

solve (2.16)–(2.19). We see in this case that the principal orbits are again $S^3\times S^3$ and the singular orbit $\lbrace 0 \rbrace \times S^3$ is associative.

In this setting, the geometry at infinity presents a new feature (that also exists in the BB manifolds below): there is a circle that remains of finite length at infinity. More precisely, the metric is asymptotic to a metric on a circle bundle over a 6-dimensional cone with the fibres of the fibration having constant finite length. The length of this circle is the limit of $A_1$ at infinity: for the family depending on the parameters $\lambda , c$ this is $2c\lambda /3$. One also sees that the volume of the associative $S^3$ is $B_1^{\lambda }(c\lambda ^2)B_2^{\lambda }(c\lambda ^2)^2 \sim (c \lambda )^3$, and so, using this family, it is impossible to vary the size of the circle while keeping the volume of the singular orbit fixed.

In [3], Bogoyavlenskaya constructed a 1-parameter family (up to scaling) of $\mathrm{SU}(2)^2\times U(1)$-invariant, cohomogeneity-1, $G_2$-holonomy metrics on $\mathbb {R}^4\times S^3$, obtained by continuously deforming the BGGG metric. With these metrics, one can independently vary the size of the circle at infinity and the associative $S^3$, and thus, in particular, obtain the BS metric as a limit of the family.

The BGGG metric is the only one from [3] which is explicitly known. Choosing the scaling so that the circle at infinity has size 1, for large *t* we compute that $t(r) \sim r$, so

$$\begin{aligned} A_1 = 1 +O(t^{-2}),\,\, A_2 = \frac{t}{\sqrt{3}} +O(t^{-1}), \,\, B_1 = \frac{2t}{3} + O(t^0),\, \ B_2 = \frac{t}{\sqrt{3}} + O(t^{-1}), \end{aligned}$$

and thus the metric is asymptotic to

$$\begin{aligned} h=dt^2 + 4(\eta _1^+ )^2 + \frac{4t^2}{3} \left( (\eta _2^+ )^2 + ( \eta _3^+)^2 \right) + \frac{16t^2}{9}(\eta _1^- )^2 + \frac{4t^2}{3} \left( (\eta _2^- )^2 + (\eta _3^-)^2 \right) . \end{aligned}$$

This limit of the family of metrics given by (2.24), (2.25) as $c\rightarrow 0$ is an $S^1$-bundle over a Calabi–Yau cone on the standard homogeneous Sasaki–Einstein metric on $S^2\times S^3$. This conical Calabi–Yau metric is also known as the conifold or 3-dimensional ordinary double point.

#### The Bazaikin–Bogoyavlenskaya (BB) metrics

The Bazaikin–Bogoyavlenskaya $G_2$-manifolds *X* [1] (BB manifolds for short) have group diagram $I(\mathrm{SU}(2)^2; \mathbb {Z}_4 ; U(1))$, i.e. the principal orbits are of the form $S^3 \times S^3/\mathbb {Z}_4$ and the (unique) singular orbit is $\mathrm{SU}(2)^2/U(1) \cong S^2 \times S^3$. In fact, *X* is diffeomorphic to $L^4 \times S^3$, where $L \rightarrow S^2$ is the complex line bundle canonically associated with the Hopf bundle and $L^4$ denotes its fourth tensor power.

In [1] some complete torsion-free $G_2$-structures with an extra *U*(1)-symmetry, i.e. with $A_2=A_3$ and $B_2=B_3$, are constructed. These structures give rise to a 1-parameter family of holonomy $G_2$-metrics on $X=L^4 \times S^3$, which have $(A_1(0),A_2(0),B_1(0), B_2(0))= ( \mu , \lambda ,0 , \lambda )$ for some values of $\lambda , \mu \in \mathbb {R}$ with $\lambda ^2 + \mu ^2 =1$. In particular, the volume of the singular orbit $S^2 \times S^3$ is proportional to $\lambda ^4 \mu =(1-\mu ^2)^2 \mu$ and that of any 3-sphere $*\times S^3$ is proportional to $\lambda ^2 \mu = (1-\mu ^2)\mu$: these 3-spheres are *not* associative. At least some of these metrics are asymptotic to an $S^1$-bundle over the conifold.

We have some preliminary results on $G_2$-instantons on these $G_2$-manifolds and intend to investigate them further in future work.

##### Remark 2

The examples of $G_2$-manifolds in Sects. 2.2.2–2.2.3 are asymptotic at infinity to a circle bundle over a cone: such manifolds are called asymptotically locally conical (ALC), and it is well-known that the asymptotic cone is Calabi–Yau. One can see, under suitable assumptions, that $G_2$-instantons on ALC $G_2$-manifolds are asymptotic to Calabi–Yau monopoles on the Calabi–Yau cone. See [21] for some examples and results on Calabi–Yau monopoles in the asymptotically conical and conical settings.

### Homogeneous bundles and invariant fields

We will now classify invariant connections on bundles over the SU(2)$^2$-principal orbits in the $G_2$-manifolds *X* of Sect. 2.2 so $X\cong \mathbb {R}^4\times S^3$ or $L^4\times S^3$.

We start with a review of the general setup on a homogeneous manifold *K* / *H*. First, *K*-homogeneous *G*-bundles over *K* / *H* (which will be our principal orbits) are determined by their isotropy homomorphism. These are group homomorphisms $\lambda : H \rightarrow G$, associated with which we construct the bundle $P_{\lambda }=K \times _{(H,\lambda )} G$. The reductive splitting $\mathfrak {k} = \mathfrak {h} \oplus \mathfrak {m}$ equips $K \rightarrow K/H$ with a connection whose horizontal space is $\mathfrak {m}$. This is the so-called canonical invariant connection and its connection form $A^c_{\lambda } \in \Omega ^1(K, \mathfrak {g})$ is the left-invariant translation of $d \lambda \oplus 0 : \mathfrak {h} \oplus \mathfrak {m} \rightarrow \mathfrak {g}$. Other invariant connections are classified by Wang’s theorem [28] and are in correspondence with morphisms of *H*-representations $\Lambda : (\mathfrak {m} , {{\mathrm{Ad}}}) \rightarrow (\mathfrak {g} , {{\mathrm{Ad}}}\circ \lambda )$.

In the cases we shall consider, SU(2)$^2$ acts with cohomogeneity-1 and the principal orbits are of the form $M=S^3 \times S^3 / H$, where *H* will only be nontrivial in the BB case where it is $\mathbb {Z}^4$. Isomorphism classes of homogeneous *G*-bundles on these principal orbits are in correspondence with (conjugacy classes) of isotropy homomorphisms, i.e. group homomorphisms $\lambda : H \rightarrow G$. Therefore $\lambda$ will be the trivial homomorphism, except in the BB case where the possible $\lambda$’s are in one-to-one correspondence with cyclic subgroups of *G* of order 1, 2 or 4. Given such $\lambda$ determines the SU(2)$^2$-homogeneous *G*-bundle

$$\begin{aligned} P_\lambda = \mathrm{SU}(2)^2 \times _{(H,\lambda )} G. \end{aligned}$$

The canonical invariant connection $a_c$ is the trivial one (given the choice of *H*), hence its connection 1-form as an element of $\Omega ^1(\mathrm{SU}(2)^2, \mathfrak {g})$ vanishes. It follows from Wang’s theorem [28], that any other invariant connection differs from $a_c$ by a morphism of *H*-representations

$$\begin{aligned} \Lambda : ( \mathfrak {su}^+ \oplus \mathfrak {su}^- , {{\mathrm{Ad}}}) \rightarrow ( \mathfrak {g} , {{\mathrm{Ad}}}\circ \lambda ). \end{aligned}$$

When *H* is trivial, both these representations are trivial, and so $\Lambda$ is any linear map. Given such a $\Lambda$ we extend it by left-invariance to SU(2)$^2$. This gives rise to the 1-form with values in $\mathfrak {g}$:

2.27 $$\begin{aligned} a=\sum _{i=1}^3 a_i^+ \otimes \eta _i^+ + a_i^- \otimes \eta _i^-, \end{aligned}$$

where $a_i^{\pm } \in \mathfrak {g}$ are constant on each principal orbit. Hence, on the open dense set $\mathbb {R}^+_t \times M \subset X$, the most general $\mathrm{SU}(2) \times \mathrm{SU}(2)$-invariant connection on any $P_{\lambda }$ can be written as in (2.27) with the $a_i^{\pm }$ depending on $t \in \mathbb {R}^+$ and taking values in $\mathfrak {g}$.

#### Remark 3

We can always use an SU(2)$^2$-invariant gauge transformation $g : \mathbb {R}^+ \rightarrow G$ to put any invariant connection *A* in temporal gauge. This amounts to solving the ODE $\dot{g}g^{-1} + gA(\partial _t)g^{-1} =0$, which has a unique solution *g* converging to 1 as $t \rightarrow + \infty$.

#### Lemma 2

The curvature of the connection *a*(*t*) above on $\lbrace t \rbrace \times M$ is given by

$$\begin{aligned} F_a= & {} \sum _{i=1}^3 \left[ a_i^+, a_i^-\right] \otimes \eta _i^+ \wedge \eta _i^- \\&+\sum _{i=1}^3 \left( \left( -2 a_i^+ + \left[ a_j^+, a_k^+\right] \right) \otimes \eta _{j}^+\wedge \eta _{k}^+ + \left( -2 a_i^+ + \left[ a_j^-, a_k^-\right] \right) \otimes \eta _{j}^-\wedge \eta _{k}^- \right) \\&+ \sum _{i=1}^3 \left( \left( -2 a_i^- + \left[ a_j^-, a_k^+\right] \right) \otimes \eta _{j}^- \wedge \eta _{k}^+ + \left( -2 a_i^- + \left[ a_j^+, a_k^-\right] \right) \otimes \eta _{j}^+ \wedge \eta _{k}^- \right) , \end{aligned}$$

where in the summation above (*j*, *k*) is such that (*i*, *j*, *k*) is a cyclic permutation of (1, 2, 3).

#### Proof

We can compute the curvature via $F_a = da + \frac{1}{2}[a \wedge a]$ and the Maurer–Cartan relations (2.8), (2.9) for the coframing $\eta _i^{\pm }$. The details are a lengthy but straightforward computation. $\square$

### The SU(2)$^2$-invariant ODEs

We may now write down the ODEs arising from Eqs. (2.5) and (2.6) which describe our invariant $G_2$-instantons.

#### Lemma 3

Let (*i*, *j*, *k*) denote cyclic permutations of (1, 2, 3). Using the notation from (2.13) and (2.27), the evolution Eqs. (2.5)–(2.6) for SU(2)$^2$-invariant $G_2$-instantons *a* on $\mathbb {R}^+_t \times M$ are

$$\begin{aligned} \frac{B_i}{A_i} \dot{a}_i^+ + \left( \frac{B_i}{B_jB_k} - \frac{B_i}{A_jA_k} \right) a_i^+= & {} \frac{B_i }{2 B_j B_k } \left[ a_j^- , a_k^-\right] -\frac{B_i}{2A_j A_k} \left[ a_j^+ , a_k^+\right] ,\\ \frac{A_i}{B_i} \dot{a}_i^- + \left( \frac{A_i}{B_jA_k} + \frac{A_i}{A_jB_k} \right) a_i^-= & {} \frac{A_i }{2 B_j A_k } \left[ a_j^- , a_k^+\right] +\frac{A_i}{2A_j B_k} \left[ a_j^+ , a_k^-\right] , \end{aligned}$$

together with the constraint

$$\begin{aligned} \sum _{i=1}^3 \frac{1}{A_iB_i} \left[ a_i^+ , a_i^-\right] =0. \end{aligned}$$

#### Proof

The proof amounts to inserting the formula for the curvature $F_a$ from Lemma 2 into (2.5), (2.6). For this we need to use the $\mathrm{SU}(3)$-structure on the principal orbits given in (2.10)–(2.12). For convenience we shall write $\eta ^{\pm }_{a\cdots b}=\eta ^{\pm }_a\wedge \cdots \wedge \eta ^{\pm }_{b}$.

We start by computing

$$\begin{aligned} F_a \wedge \Omega _2= & {} -8 B_1 \left( A_2 B_3 \left( \left[ a_2^-, a_3^+\right] - 2a_1^-\right) +A_3 B_2 \left( \left[ a_2^+,a_3^-\right] -2a_1^-\right) \right) \eta _{123}^- \wedge \eta _{23}^+ \\&-8 A_1 \left( A_2 A_3 \left( \left[ a_2^-, a_3^-\right] - 2a_1^+\right) - B_2 B_3 \left( \left[ a_2^+,a_3^+\right] -2a_1^+\right) \right) \eta _{123}^+ \wedge \eta _{23}^-\\&+ \text { cyclic permutations}. \end{aligned}$$

Moreover, since $\vert \eta _i^-\vert _t = \frac{1}{2B_i}$ and $\vert \eta _i^+ \vert _t = \frac{1}{2A_i}$, we conclude that

$$\begin{aligned} *_t \left( 8\eta _{123}^- \wedge \eta _{23}^+\right) = -\frac{1}{2}\frac{A_1}{ A_2A_3B_1B_2B_3}\eta _1^+, \quad *_t \left( 8\eta _{123}^+ \wedge \eta _{23}^-\right) = \frac{1}{2}\frac{B_1}{ B_2B_3A_1A_2A_3}\eta _1^- \end{aligned}$$

and cyclic permutations. Combining this with the previous computation we obtain

$$\begin{aligned} *_t (F_a \wedge \Omega _2)= & {} \frac{1}{2} \left( \frac{A_1}{A_3B_2 } \left( \left[ a_2^-, a_3^+\right] - 2a_1^-\right) + \frac{A_1}{A_2B_3 } \left( \left[ a_2^+,a_3^-\right] -2a_1^-\right) \right) \eta _{1}^+ \\&- \frac{1}{2} \left( \frac{B_1}{B_2 B_3} \left( \left[ a_2^-, a_3^-\right] - 2a_1^+\right) - \frac{B_1}{A_2 A_3} \left( \left[ a_2^+,a_3^+\right] -2a_1^+\right) \right) \eta _{1}^- \\&+\,\, \text {cyclic permutations}. \end{aligned}$$

The complex structure $J_t$ is such that

$$\begin{aligned} J_t \eta _i^+= & {} -*\left( \eta _i^+\wedge \frac{\omega ^2}{2}\right) = *\left( 16A_jB_jA_kB_k\eta _{123}^+\eta _{jk}^-\right) = \frac{B_i}{A_i} \eta _i^- \end{aligned}$$

and so it is straightforward to compute

$$\begin{aligned} J_t \dot{a} = \sum _{i=1}^3 \frac{B_i}{A_i} \dot{a}_i^+ \otimes \eta _i^- - \frac{A_i}{B_i} \dot{a}_i^- \otimes \eta _i^+. \end{aligned}$$

Inserting our formulae in (2.7) gives the ODEs in the statement. We finally compute

$$\begin{aligned} F_a \wedge \frac{\omega ^2}{2}= -16 A_1A_2 B_1B_2 [a_3^+,a_3^-] \eta _{123}^+ \wedge \eta _{123}^-+ \text {cyclic permutations}, \end{aligned}$$

yielding the constraint in the statement. $\square$

### Elementary solutions

In this subsection we consider elementary cases of SU(2)$^2$-invariant $G_2$-instanton equations on any of the SU(2)$^2$-invariant $G_2$-manifolds of cohomogeneity-1 described in Sect. 2.2. We will let *X* denote such a $G_2$-manifold.

We verify that flat connections satisfy our $G_2$-instanton equations in Sect. 2.5.1 and we classify and describe all abelian $G_2$-instantons explicitly in Sect. 2.5.2.

#### Flat connections

Any flat connection on *X* is obviously a $G_2$-instanton and so must be a solution to our equations (for any gauge group *G*). As the fundamental group $\pi _1(X)$ is trivial, any flat connection in this setting is gauge equivalent to the trivial connection. However, on a homogeneous bundle there may be invariant flat connections that are not gauge equivalent to the trivial connection through invariant gauge transformations.

Let $A=a(t)$ be an invariant connection on *X* given as in (2.27) for $a_i^{\pm }:\mathbb {R}^+\rightarrow \mathfrak {g}$ for $i=1,2,3$. From the formula in Lemma 2 for the curvature of *a*(*t*), one sees that *A* is flat if and only if $a_i^{\pm }$ are *t*-independent and, for all cyclic permutations (*i*, *j*, *k*) of (1, 2, 3), we have

$$\begin{aligned} \left[ a_i^+, a_i^-\right]= & {} 0,\quad a_i^+ = \frac{1}{2} \left[ a_j^+, a_k^+\right] = \frac{1}{2} \left[ a_j^-, a_k^-\right] \quad \text {and}\\ \nonumber a_i^-= & {} \frac{1}{2} \left[ a_j^-, a_k^+\right] = \frac{1}{2} \left[ a_j^+, a_k^-\right] . \end{aligned}$$

It is elementary to verify that a constant (i.e. *t*-independent) choice of $a_i^{\pm }$ satisfying these conditions then solves the ODE system for SU(2)$^2$-invariant $G_2$-instantons in Lemma 3.

#### Abelian instantons

On circle bundles, equivalently complex line bundles, the Lie algebra structure of the gauge group is trivial and the $G_2$-instanton equations in Lemma 3 become linear. Consequently, it is then easy to integrate them, which we shall now proceed to do.

By Lemma 3, to find a $G_2$-instanton in this setting we must integrate

2.28 $$\begin{aligned} \dot{a}_i^+ = - \left( \frac{A_i}{B_jB_k} - \frac{A_i}{A_jA_k} \right) a_i^+, \quad \dot{a}_i^- = - \left( \frac{B_i}{B_jA_k} + \frac{B_i}{A_jB_k} \right) a_i^-. \end{aligned}$$

Given $t_0 \in \mathbb {R}^+$, the Eq. (2.28) can be integrated to

2.29 $$\begin{aligned} a_i^+ (t)= & {} a_{i}^+(t_0) \exp \left( - \int _{t_0}^t \left( \frac{A_i}{B_jB_k} - \frac{A_i}{A_jA_k} \right) ds \right) , \end{aligned}$$

2.30 $$\begin{aligned} a_i^- (t)= & {} a_{i}^-(t_0) \exp \left( - \int _{t_0}^t \left( \frac{B_i}{B_jA_k} + \frac{B_i}{A_jB_k} \right) ds \right) . \end{aligned}$$

We see from the free constants in (2.29), (2.30) that there is a family of $G_2$-instantons parametrized by 6 real parameters defined on the complement of the singular orbit in *X*. Using the results in Appendix A we can characterise the subspaces of this family of $G_2$-instantons which smoothly extend over the singular orbit in the BS, BGGG and Bogoyavlenskaya metrics.

##### Proposition 2

Let *A* be an SU(2)$^2$-invariant $G_2$-instanton on a *U*(1)-bundle, or equivalently a complex line bundle, over $\mathbb {R}^4\times S^3$ with an $\mathrm{SU}(2)^2\times U(1)$-invariant $G_2$-holonomy metric. Then *A* can be written as

$$\begin{aligned} A= \sum _{i=1}^3 a_{i}^+(t_0) \exp \left( - \int _{t_0}^t \left( \frac{A_i}{B_jB_k} - \frac{A_i}{A_jA_k} \right) ds \right) \eta _i^+ , \end{aligned}$$

for some $t_0\in \mathbb {R}^+$ and $a_i^+(t_0) \in \mathbb {R}$ for $i=1,2,3$, where (*i*, *j*, *k*) is a cyclic permutation of (1, 2, 3).

##### Proof

The principal orbits on $\mathbb {R}^4\times S^3$ are $S^3 \times S^3$ and the singular one is $S^3=\mathrm{SU}(2)^2/ \Delta \mathrm{SU}(2)$. The extensions of a circle bundle *P* on $\mathbb {R}^+\times S^3\times S^3$ to $S^3$ are parametrized by isotropy homomorphisms $\lambda :\Delta \mathrm{SU}(2)\rightarrow U(1)$. The only such homomorphism $\lambda$ is the trivial one, so the unique extension of *P* to the singular orbit is as the trivial bundle.

The canonical invariant connection on the trivial homogeneous bundle vanishes as an element of $\Omega ^1(\mathrm{SU}(2)^2 , \mathbb {R})$. Any other invariant connection on this bundle is then given as an element of $\Omega ^1(\mathrm{SU}(2)^2 , \mathbb {R})$ by the pullback of a bi-invariant 1-form on $S^3=\mathrm{SU}(2)^2/ \Delta \mathrm{SU}(2)$. However, the only such 1-form is the zero form, so the connection *A* extends over the singular orbit if and only if Lemma 9 in Appendix A applies to the 1-form $a=\sum _{i=1}^3 a_i^+ \eta _i^+ + a_i^- \eta _i^-$.

We deduce that, for *t* near 0, the $a_{i}^{\pm }(t)$ are even and $a_i^{\pm }(0)=0$ for $i=1,2,3$. We know by Appendix A (and explicitly by Examples 1–2 for the BS and BGGG metrics) that for *t* near 0 we have $A_i(t)= \frac{t}{2} + t^3 C_i(t)$ and $B_i(t)=b_0+t^2 D_i(t)$, for some real analytic $C_i$, $D_i$ and some constant $b_0\ne 0$. Then, choosing $0<t_0 \ll 1$ and using the expressions (2.29), (2.30), we compute that for $t < t_0 \ll 1$

$$\begin{aligned} a_i^+(t) = a_i^+(t_0)t_0^{-2} t^2 + \cdots \quad \text {and} \quad a_i^-(t) = a_i^-(t_0) t_0^4 t^{-4} + O(1). \end{aligned}$$

Applying Lemma 9 to *a*, we deduce that $a_i^-(t_0)$ must vanish for $i=1,2,3$, while the $a_i^+(t_0)$ can be freely chosen. $\square$

In the BS or BGGG case, we can evaluate the integrals in Proposition 2 to give the following.

##### Corollary 1

Let *A* be an SU(2)$^2$-invariant $G_2$-instanton with gauge group *U*(1) over the BS or BGGG $G_2$-manifold $\mathbb {R}^4\times S^3$ described in Sect. 2.2.

- In the BS case, *A* can be written as
  $$\begin{aligned} A =\frac{r^3-1}{r}\sum _{i=1}^3x_i\eta _i^+ \end{aligned}$$
  for some $x_1,x_2,x_3\in \mathbb {R}$, where $r\in [1,+\infty )$ is determined by (2.22).
- In the BGGG case, *A* can be written as
  $$\begin{aligned} A=\frac{(r-9/4)(r+9/4)}{(r-3/4)(r+3/4)}x_1\eta _1^++ \frac{(r-9/4)e^r}{\sqrt{r}(r+9/4)^2}\left( x_2\eta _2^++x_3\eta _3^+\right) \end{aligned}$$
  for some $x_1,x_2,x_3\in \mathbb {R}$, where $r\in [9/4,+\infty )$ is given by (2.26). When $x_2=x_3=0$, *A* is a multiple of the harmonic 1-form dual to the Killing field generating the *U*(1)-action.

We already observe a marked difference in the behaviour of $G_2$-instantons on the BS and BGGG $\mathbb {R}^4\times S^3$ in this simple abelian setting. In particular, the instantons in the BS case all have bounded curvature, whereas those in the BGGG case have bounded curvature only when $x_2=x_3=0$, in which case the curvature also decays to 0 as $r\rightarrow \infty$.

##### Remark 4

Of course, for any abelian gauge group all Lie brackets vanish and the ODE system decouples into several independent linear ODEs for instantons on circle bundles. Hence, the construction of abelian $G_2$-instantons here reduces to the *U*(1) case given in Proposition 2.

## $\mathrm{SU}(2)^3$-invariant $G_2$-instantons

In the Bryant–Salamon case the torsion-free $G_2$-structure is described in Sect. 2.2.1. Recall that the structure enjoys an extra SU(2)-symmetry, so that $A_1=A_2=A_3$ and $B_1=B_2=B_3$ where

3.1 $$\begin{aligned} \dot{A}_1=\frac{1}{2} \left( 1- \frac{A_1^2}{B_1^2} \right) , \quad \dot{B}_1= \frac{A_1}{B_1}. \end{aligned}$$

We shall use this notation throughout this section.

The only possible homogeneous SU(2)-bundle *P* on the principal orbits $S^3\times S^3$ is $P=\mathrm{SU}(2)^2 \times \mathrm{SU}(2)$, i.e. the trivial SU(2)-bundle. We consider connection 1-forms with the extra SU(2)-symmetry existent in the underlying geometry.

We begin in Sect. 3.1 by simplifying the ODEs and constraint system in Lemma 3 to this more symmetric situation, and then derive the conditions necessary to extend the solution to this system across the singular orbit in Sect. 3.2. We give classification results for the solutions to these equations in Sect. 3.3. We also examine the asymptotic behaviour of the solutions in terms of a connection on $S^3\times S^3$, and give a compactness result for the space of solutions. The latter result is related to the familiar “bubbling” and “removable singularities” phenomena.

### The SU(2)$^3$-invariant ODEs

We simplify the invariant $G_2$-instanton equations from Lemma 3 in this setting.

#### Proposition 3

Let *A* be an $\mathrm{SU}(2)^3$-invariant $G_2$-instanton with gauge group $\mathrm{SU}(2)$ on $\mathbb {R}^+ \times \mathrm{SU}(2)^2 \cong \mathbb {R}^+ \times \mathrm{SU}(2)^3/ \Delta \mathrm{SU}(2)$. There is a standard basis $\{T_i\}$ of $\mathfrak {su}(2)$, i.e. with $[T_i , T_j]= 2 \epsilon _{ijk} T_k$, such that (up to an invariant gauge transformation) we can write

3.2 $$\begin{aligned} A=A_1x\left( \sum _{i=1}^3 T_i\otimes \eta _i^+ \right) + B_1y\left( \sum _{i=1}^3 T_i\otimes \eta _i^- \right) , \end{aligned}$$

with *x*, $$y:\mathbb {R}^+\rightarrow \mathbb {R}$$ satisfying

3.3 $$\begin{aligned} \dot{x}&= \frac{\dot{A}_1}{A_1} x + y^2-x^2 \quad = \frac{1}{2A_1}\left( 1-\frac{A_1^2}{B_1^2}\right) x+y^2-x^2,\end{aligned}$$

3.4 $$\begin{aligned} \dot{y}&= \frac{ 2\dot{A}_1 -3}{A_1} y +2xy \quad = -\frac{1}{A_1}\left( 2+\frac{A_1^2}{B_1^2}\right) y+2xy. \end{aligned}$$

#### Proof

We start by realizing SU(2)$^2$ as $\mathrm{SU}(2)^3/\Delta \mathrm{SU}(2)$. Isomorphism classes of $\mathrm{SU}(2)^3$-equivariant bundles over SU(2)$^2$ are then in correspondence with conjugation classes of homomorphisms $\mu : \mathrm{SU}(2) \rightarrow \mathrm{SU}(2)$. There are only two such conjugation classes, namely those represented by the identity and the trivial homomorphism.

We begin with the case where $\mu$ is the identity. First, we fix a reductive decomposition of $\mathfrak {su}(2)^3$, i.e. a complement $\mathfrak {m}$ of the isotropy algebra $\Delta \mathfrak {su}(2) \subset \mathfrak {su}(2)^3$ such that $[\Delta \mathfrak {su}(2) , \mathfrak {m}] \subset \mathfrak {m}$. We set $\Delta ^+,\Delta ^- \subset \mathfrak {su}^2$ to be diagonal and anti-diagonal respectively, and let

$$\begin{aligned} \mathfrak {m} = (0 \oplus \Delta ^+ ) \oplus (0 \oplus \Delta ^-). \end{aligned}$$

By Wang’s theorem [28], any SU(2)$^3$-invariant connection can be written as

$$\begin{aligned} A= d \mu + \Lambda ^+ + \Lambda ^- \in \Omega ^1(\mathrm{SU}(2)^3 , \mathfrak {su}(2)), \end{aligned}$$

where $\Lambda ^{\pm }: ( \Delta ^{\pm }, {{\mathrm{Ad}}}) \rightarrow (\mathfrak {su}(2), {{\mathrm{Ad}}}\circ \mu )$ are morphisms of SU(2)-representations. We now pull *A* back to SU(2)$^2$ via the map $\psi : \mathrm{SU}(2)^2 \rightarrow \mathrm{SU}(2)^3$ given by $\psi (g_1,g_2)= (g_2 g_1^{-1} , g_1 , g_2)$. Then $\psi ^* d \mu =0$ and $\psi ^* \Lambda ^{\pm } = f^{\pm }_{ij} T_j \otimes \eta _i^{\mp }$ (the inversion to $\mp$ on the $\eta _i^{\mp }$ is correct!), for some functions $f_{ij}^{\pm }$ and fixed standard basis $\{T_i\}$ of $\mathfrak {su}(2)$. Extending naturally to $\mathbb {R}^+ \times \mathrm{SU}(2)^2$ we obtain $a_i^{\pm }=f^{\mp }_{ij} T_j$.

For a fixed $t\in \mathbb {R}^+$, we can apply a gauge transformation so that $\mu ={{\mathrm{id}}}$. Hence, we can write $a_i^+(t)=A_1(t) x(t)$ and $a_i^-(t)=B_1(t) y(t)$ for $i=1,2,3$, since the adjoint representation of SU(2) is irreducible, where we have introduced the non-zero factors of $A_1$ and $B_1$ for convenience. Since the gauge transformation depends on *t*, we deduce that we can write

$$\begin{aligned} A=A_1x \gamma \left( \sum _i T_i\otimes \eta _i^+\right) \gamma ^{-1} +B_1y \gamma \left( \sum _i T_i\otimes \eta _i^-\right) \gamma ^{-1} \end{aligned}$$

for some functions $\gamma :\mathbb {R}^+\rightarrow \mathrm{SU}(2)$ and $x,y:\mathbb {R}^+\rightarrow \mathbb {R}$.

We now turn to the ODEs and constraint from Lemma 3 arising from the $G_2$-instanton condition. We see that the constraint is immediately satisfied and the symmetry in the ODEs forces

$$\begin{aligned} A_1x[\gamma ^{-1}\dot{\gamma },T_i]=0\quad \text {and}\quad B_1y[\gamma ^{-1}\dot{\gamma },T_i]=0 \end{aligned}$$

for $i=1,2,3$, which means $\dot{\gamma }=0$ if *A* is non-zero. Therefore, we may write *A* as in (3.2). Using (3.1), we conclude that the ODEs from Lemma 3 imply that *x* and *y* satisfy (3.3)–(3.4) as claimed.

We turn now to the case when $\mu : \mathrm{SU}(2) \rightarrow \mathrm{SU}(2)$ is the trivial homomorphism. Here, the canonical invariant connection $d \mu$ vanishes as a 1-form on $\mathrm{SU}(2)^3$ with values in $\mathfrak {su}(2)$. By Wang’s theorem, any other invariant connection is then given by a morphism of $\Delta \mathrm{SU}(2)$-representations $\Lambda : ( \mathfrak {m}, {{\mathrm{Ad}}}) \rightarrow (\mathfrak {su}(2) , {{\mathrm{Ad}}}\circ \mu )$. The left-hand side splits into two copies of the adjoint representation of $\mathrm{SU}(2)$ while the right-hand side decomposes into three trivial representations. Schur’s lemma then implies that $\Lambda$ must vanish and so the trivial connection is the unique invariant one on this homogeneous bundle. This corresponds to taking $x=y=0$ in the statement. $\square$

### Initial conditions

Now we determine the initial conditions in order for an $\mathrm{SU}(2)^3$-invariant $G_2$-instanton *A*, given by a solution to the ODEs in Proposition 3, to extend smoothly over the singular orbit $S^3=\mathrm{SU}(2)^2/ \Delta \mathrm{SU}(2)$. For that we need to first extend the bundle over the singular orbit. Up to an isomorphism of homogeneous bundles, there are two possibilities: these are

3.5 $$\begin{aligned} P_{\lambda }=\mathrm{SU}(2)^2 \times _{(\Delta \mathrm{SU}(2), \lambda )} \mathrm{SU}(2), \end{aligned}$$

with the homomorphism $\lambda :\mathrm{SU}(2)\rightarrow \mathrm{SU}(2)$ being either the trivial one (which we denote by 1) or the identity ${{\mathrm{id}}}$. Depending on the choice of $\lambda$, the conditions for the connection *A* to extend are different, as we show in the following lemma.

#### Lemma 4

The connection *A* in (3.2) extends smoothly over the singular orbit $S^3$ if *x*(*t*) is odd, *y*(*t*) is even, and their Taylor expansions around $t=0$ are

- either $x(t)=x_1t+ x_3 t^3+\cdots , \ y(t)= y_2 t^2 + \cdots$, in which case *A* extends smoothly as a connection on $P_{1}$;
- or $x(t)=\frac{2}{t}+x_1t+\cdots , \ y(t)=y_0 + y_2 t^2 +\cdots$, in which case *A* extends smoothly as a connection on $P_{{{\mathrm{id}}}}$.

#### Proof

We only analyze the case $\lambda ={{\mathrm{id}}}$ in detail, as both situations are similar.

When $\lambda ={{\mathrm{id}}}$, the canonical invariant connection associated with the reductive splitting $\mathfrak {su}(2)^2= \mathfrak {su}^+(2) \oplus \mathfrak {su}^-(2)$ is

3.6 $$\begin{aligned} A^{\text {can}}= \sum _{i=1}^3 T_i \otimes \eta _i^+ \in \Omega ^1(\mathrm{SU}(2)\times \mathrm{SU}(2), \mathfrak {su}(2)). \end{aligned}$$

Therefore, for *A* to extend over the singular orbit as a connection on $P_{{{\mathrm{id}}}}$ we need to apply Lemma 10 in Appendix A to the 1-form

$$\begin{aligned} A-A^{\text {can}}= \left( A_1x - 1 \right) \left( \sum _{i=1}^3 T_i\otimes \eta _i^+ \right) + B_1y \left( \sum _{i=1}^3 T_i\otimes \eta _i^- \right) . \end{aligned}$$

We conclude that *A* extends over the singular orbit $S^3$ if

- $A_1(t)x(t)$, $B_1(t)y(t)$ are both even,
- $\lim _{t \rightarrow 0} A_1(t)x(t)=1$ and $\lim _{t\rightarrow 0}B_1(t)y(t)$ is finite.

By Lemma 8 in Appendix A (or by inspection since the BS metric is explicit), we see that $A_1(t)$ is odd and $B_1(t)$ is even, so *x*(*t*) and *y*(*t*) must be odd and even respectively. Moreover, $\dot{A}_1(0)=\frac{1}{2}$ and $B_1(0)\ne 0$, as we see in Example 1 in Appendix A, so the expansions of *x*, *y* around zero must be as claimed in the lemma.

To carry over the analysis in the case where $\lambda =1$ we apply Lemma 10 directly to the 1-form *A*, giving $A_1x$, $B_1y$ are even with $\lim _{t\rightarrow 0}A_1x=\lim _{t\rightarrow 0}B_1y=0$. $\square$

### Solutions and their properties

We now describe solutions of the $\mathrm{SU}(2)^3$-invariant $G_2$-instanton equations, which splits into two cases: when the bundle $P=P_1$ and when $P=P_{{{\mathrm{id}}}}$, in the notation of the previous subsection. In the first case we recover the $G_2$-instantons constructed in [8], and in the second case we find a new example of a $G_2$-instanton. We then analyse the asymptotic behaviour of the instantons, and finally show that the $\mathbb {R}_{\ge 0}$-family of solutions on $P_1$ admits a natural compactification.

#### Solutions smoothly extending on $P_1$

Clarke [8] constructed a 1-parameter family of $G_2$-instantons on the Bryant–Salamon $\mathbb {R}^4\times S^3$. These instantons live on the bundle $P_1$ given by (3.5), i.e. when the homomorphism $\lambda$ is trivial. Moreover, they have $y=0$ in the notation of Proposition 3, and so the ODEs there reduce to a single ODE for *x* which can be explicitly integrated. We shall reconstruct these $G_2$-instantons in the proof of the next result, which classifies and explicitly describes the $G_2$-instantons that smoothly extend over the singular orbit on the bundle $P_1$.

##### Theorem 4

Let *A* be an $\mathrm{SU}(2)^3$-invariant $G_2$-instanton with gauge group $\mathrm{SU}(2)$ on the Bryant–Salamon $G_2$-manifold $\mathbb {R}^4\times S^3$, which smoothly extends over the singular orbit on $P_{1}$. Then, *A* is one of Clarke’s examples [8], in which case there is $x_1 \in \mathbb {R}$ such that, in the notation of Proposition 3,

3.7 $$\begin{aligned} x(t)=\frac{2x_1 A_1(t)}{1+ x_1 \left( B_1^2(t)-\frac{1}{3}\right) }\quad \text {and}\quad y(t)=0. \end{aligned}$$

Given such an $x_1 \in \mathbb {R}$ we shall denote the resulting instanton by $A^{x_1}$. Observe that $A^{x_1}$ is defined globally on $\mathbb {R}^4\times S^3$ if and only if $x_1\ge 0$ and that $A^0$ is the trivial flat connection.

##### Proof

It will be enough to show that any instanton as in the statement defined on a neighbourhood of the singular orbit must coincide with one of Clarke’s examples there. For that, let (*x*(*t*), *y*(*t*)) be a solution to the ODEs (3.3), (3.4). We shall show that if the resulting instanton *A* extends over the singular orbit then $y(t)=0$ for all *t*.

Recall from Lemma 4 that for *A* to smoothly extend over the singular orbit on $P_1$ we must have

$$\begin{aligned} x(t) = x_1 t + t^3 u(t)\quad \text {and}\quad y(t) = t^2 v(t) \end{aligned}$$

for *t* near 0, where *u*, *v* are real analytic even functions of *t*. The system (3.3), (3.4) for *x*, *y* becomes the following system for *u*, *v*:

3.8 $$\begin{aligned} \dot{u}= & {} - \frac{2u + x_1^2 + x_1/2 }{t} + f_1(t,u,v), \end{aligned}$$

3.9 $$\begin{aligned} \dot{v}= & {} - \frac{ 6v}{t} + f_2(t,u,v), \end{aligned}$$

where $f_1,f_2: [0,+\infty ) \times \mathbb {R}^2 \rightarrow \mathbb {R}$ are some other real analytic functions. The existence and uniqueness theorem for equations with regular singular points (see [18, chapters 6 and 7], and Theorem 4.7 in [13] for a clearer statement) applies here provided that

$$\begin{aligned} u(0)=-\frac{x_1}{4}-\frac{x_1^2}{2}\quad \text {and}\quad v(0)=0. \end{aligned}$$

In that case, for each $x_1 \in \mathbb {R}$ we obtain a unique solution (*x*(*t*), *y*(*t*)) in $[0, \epsilon )$, for some $\epsilon >0$.

We are left with showing that all such solutions have $y=0$. That is indeed the case as we can simply set $y=0$ and integrate the equation for *x*:

3.10 $$\begin{aligned} \dot{x}=\frac{\dot{A}_1}{A_1} x-x^2. \end{aligned}$$

Writing this equation as

3.11 $$\begin{aligned} \frac{d}{dt}\left( \frac{x}{A_1} \right) = -A_1 \left( \frac{x}{A_1} \right) ^2 \end{aligned}$$

makes it separable. Since $B_1\dot{B}_1=A_1$ by (3.1) and $B_1^2(0)=\frac{1}{3}$, (3.11) can be readily integrated to show that *x* is given as in (3.7). By uniqueness the solutions guaranteed by the local existence theorem must be these ones and so have $y=0$. These are the $G_2$-instantons found in [8]. $\square$

Using the implicit coordinate $r\in [1,+\infty )$ in (2.22) and the formula (2.23) we can explicitly write the $G_2$-instanton $A^{x_1}=A_1xT_i\otimes \eta _i^+$ with

$$\begin{aligned} A_1(r)=\frac{r}{3}\sqrt{1-r^{-3}}\quad \text {and}\quad x(r)=\frac{2x_1r\sqrt{1-r^{-3}}}{3+x_1(r^2-1)}. \end{aligned}$$

We see that the curvature of $A^{x_1}$ is

$$\begin{aligned} F_{A^{x_1}} = T_i\otimes \left( \frac{d}{dr}(A_1x)dr\wedge \eta _i^++ A_1x(A_1x-1)\epsilon _{ijk}\eta _j^+\wedge \eta _k^+ - A_1 x \epsilon _{ijk}\eta _j^-\wedge \eta _k^- \right) . \end{aligned}$$

This can then be used to compute that

3.12 $$\begin{aligned} |F_{A^{x_1}}|^2= \frac{9}{2B_1^2} \Big \vert \frac{d}{dr}(A_1 x) \Big \vert ^2 + \frac{3x^2}{2} \frac{(A_1 x-1)^2}{A_1^2} + \frac{3A_1^2 x^2}{2 B_1^2}, \end{aligned}$$

which shows that $|F_{A^{x_1}}|$ decays at infinity at $O(r^{-2})$. Observe in particular that the curvature of $A^{x_1}$ does not lie in $L^2$.

#### Solutions smoothly extending on $P_{{{\mathrm{id}}}}$

We now turn to solutions defined on the bundle $P_{{{\mathrm{id}}}}$ given by (3.5) with the homomorphism $\lambda ={{\mathrm{id}}}$. We first give a local existence result for instantons on $P_{{{\mathrm{id}}}}$.

##### Proposition 4

Let $S^3$ be the singular orbit in the Bryant–Salamon $G_2$-manifold $\mathbb {R}^4\times S^3$. There is a one-parameter family of $\mathrm{SU}(2)^3$-invariant $G_2$-instantons, with gauge group SU(2), defined in a neighbourhood of $S^3$ and smoothly extending over $S^3$ on $P_{{{\mathrm{id}}}}$. The instantons are parametrized by $y_0 \in \mathbb {R}$ and satisfy, in the notation of Proposition 3,

$$\begin{aligned} x(t) = \frac{2}{t} + \frac{y_0^2-1}{4} t + O(t^3) , \quad y(t) = y_0 + \frac{y_0}{2} \left( \frac{y_0^2}{2} - 3 \right) t^2 + O(t^4) . \end{aligned}$$

##### Proof

We consider the initial value problem for (*x*(*t*), *y*(*t*)) to be a solution to the ODEs (3.3), (3.4) on $P_{{{\mathrm{id}}}}$. By Lemma 4, the conditions for smooth extension over the singular orbit are that

$$\begin{aligned} x(t) = \frac{2}{t} + t u(t) , \quad y(t) = y_0 + t^2 v(t), \end{aligned}$$

for some real analytic functions *u*, $v: [0, + \infty ) \rightarrow \mathbb {R}$. Substituting these expressions and the expansion of $A_1$ from Example 1 into (3.3), (3.4) yields

3.13 $$\begin{aligned} \dot{u}= & {} \frac{y_0^2-4u-1}{t} + f_1(t,u,v), \end{aligned}$$

3.14 $$\begin{aligned} \dot{v}= & {} - \frac{2v+5y_0/2-2y_0 u}{t} + f_2(t,u,v), \end{aligned}$$

where $f_1,f_2: [0,+\infty ) \times \mathbb {R}^2 \rightarrow \mathbb {R}$ are two real analytic functions up to $t=0$. Now we use the existence and uniqueness theorem for equations with regular singular points (chapters 6 and 7 in [18], or Theorem 4.7 [13]). At this stage, this requires that (*u*(0), *v*(0)) are such that the $O(t^{-1})$ terms in (3.13), (3.14) vanish and that the linear map $(u,v) \mapsto (-4 u , 2 y_0 u -2v)$ has no eigenvalues in the positive integers. The second condition holds (the eigenvalues are $-2,-4$) and the first condition requires that

$$\begin{aligned} u(0)=\frac{y_0^2-1}{4} , \quad v(0) = \frac{y_0}{2} \left( \frac{y_0^2}{2} - 3 \right) . \end{aligned}$$

The theorem for equations with regular singular points applies and shows that, under these conditions, for each $y_0 \in \mathbb {R}$ there is a unique solution (*u*(*t*), *v*(*t*)) to (3.13), (3.14), which gives the result. $\square$

##### Theorem 5

The $G_2$-instanton $A^{\lim }$ arising from the case when $y_0=0$ in Proposition 4 has

$$\begin{aligned} x(t)= \frac{A_1(t)}{\frac{1}{2}\left( B_1^2(t)-\frac{1}{3}\right) }\quad \text {and}\quad y(t)=0. \end{aligned}$$

Moreover, $A^{\lim }$ extends as a $G_2$-instanton to the Bryant–Salamon $G_2$-manifold $\mathbb {R}^4\times S^3$.

##### Proof

Back to the functions *x*, *y* in Proposition 4, we have that $y=0$ and *x* is the unique solution to

$$\begin{aligned} \dot{x}=\frac{\dot{A}_1}{A_1} x-x^2, \quad \lim _{t \rightarrow 0} A_1(t) x(t)=1. \end{aligned}$$

Writing the ODE in the form (3.11) makes it separable, and using the initial condition we obtain the solution claimed. Since *x*(*t*) is defined for all *t*, the resulting instanton is globally defined. $\square$

Again using the coordinate $r\in [1,\infty )$ in (2.22) and the formula (2.23) we can write $A^{\lim }$ explicitly with

$$\begin{aligned} x(r)=\frac{2r\sqrt{1-r^{-3}}}{r^2-1}. \end{aligned}$$

From (3.12) we see that the curvature of $A^{\lim }$ decays at infinity at order $O(r^{-2})$, just as for $A^{x_1}$.

##### Remark 5

The reader may wonder about potential $G_2$-instantons *A* arising from the local solutions with $y_0\ne 0$ in Proposition 4. Numerical investigation appears to indicate that such local solutions do not extend globally, if we impose the condition that the curvature of *A* decays at infinity. We hope to study this situation further.

#### Asymptotics of the solutions

We now consider the asymptotic behaviour of the $G_2$-instantons $A^{x_1}$ and $A^{\lim }$ constructed in Theorems 4 and 5.

Using the formula (3.7) for Clarke’s $G_2$-instanton $A^{x_1}$ we see that for $x_1>0$ and large *t*, the connection form *a*(*t*) on the time *t* slice of $\mathbb {R}^3\times S^3$, which is diffeomorphic to $S^3\times S^3$, is given by

$$\begin{aligned} a(t)= & {} \frac{2x_1 A_1^2(t)}{1+ x_1 \left( B_1^2(t)-\frac{1}{3}\right) } \sum _{i=1}^3 T_i\otimes \eta _i^+ \sim \frac{2x_1 \frac{t^2}{9}}{1+ 2x_1 \frac{t^2}{6} } \sum _{i=1}^3 T_i\otimes \eta _i^+ \\&\sim \frac{2}{3} \frac{1}{1+\frac{3}{x_1 t^2}}\sum _{i=1}^3 T_i\otimes \eta _i^+ , \end{aligned}$$

where we used the asymptotic behaviour of $A_1$, namely that $A_1(t) \sim \frac{t}{3} + O(t^{-2})$ and $B_1(t)\sim \frac{t}{\sqrt{3}}$ for *t* large. Therefore

3.15 $$\begin{aligned} a_{\infty } := \lim _{t \rightarrow +\infty }a(t)=\frac{2}{3}\sum _{i=1}^3 T_i\otimes \eta _i^+ \end{aligned}$$

is the canonical $\mathrm{SU}(2) \subset \mathrm{SU}(3)$ connection for the homogeneous nearly Kähler structure on $S^3 \times S^3$. Recall that such connections are pseudo-Hermitian–Yang–Mills (or nearly Kähler instantons). We can also compute the rate at which this happens and conclude that there is $c>0$ such that $\vert a - a_{\infty } \vert \le \frac{c}{\vert x_1 \vert t^3}$ along the end.

Similarly, we compute that for $t \gg 1$

$$\begin{aligned} A^{\lim }= & {} \frac{A_1^2(t)}{\frac{1}{2}\left( B_1^2(t)-\frac{1}{3}\right) } \sum _{i=1}^3 T_i\otimes \eta _i^+ = \frac{(t/3 + O(t^{-2}))^2}{t^2/6 + O(t^{-1})} \sum _{i=1}^3 T_i\otimes \eta _i^+\\= & {} \frac{2}{3} ( 1+ O(t^{-3}) ) \sum _{i=1}^3T_i\otimes \eta _i^+ . \end{aligned}$$

Thus, $\vert A^{\lim } - a_{\infty } \vert = O(t^{-4})$, as $\vert \eta _i^+ \vert = O(t^{-1})$. We summarize these conclusions.

##### Proposition 5

Let *A* be an $\mathrm{SU}(2)^3$-invariant $G_2$-instanton given by Theorem 4 or 5 which is defined globally on the Bryant–Salamon $G_2$-manifold $\mathbb {R}^4\times S^3$. Then *A* is asymptotic to the canonical pseudo-Hermitian–Yang–Mills connection $a_{\infty }$ in (3.15) for the homogeneous nearly Kähler structure on $S^3 \times S^3$. In particular:

- if $A=A^{x_1}$ for some $x_1 \in \mathbb {R}^+$, then for $t \gg 1$
  $$\begin{aligned} \vert A^{x_1} - a_{\infty } \vert \le \frac{c}{ x_1 t^3}, \end{aligned}$$
  where $c>0$ is some constant independent of $x_1$;
- if $A=A^{\lim }$, then for $t \gg 1$, $\vert A^{\lim } - a_{\infty } \vert = O(t^{-4})$.

##### Remark 6

As previously mentioned, any $G_2$-instanton on an asymptotically conical $G_2$-manifold which has a well-defined limit at infinity and has pointwise decaying curvature will be asymptotic to a pseudo-HYM connection on the link of the asymptotic cone [20]. Proposition 5 refines this result in this setting.

#### Compactness properties of the moduli of solutions

Next we show that as $x_1 \rightarrow + \infty$ Clarke’s $G_2$-instantons $A^{x_1}$ “bubble off” an anti-self-dual (ASD) connection along the normal bundle to the associative $S^3= \{0\}\times S^3 \subset \mathbb {R}^4\times S^3$. We shall also show that in the same limit Clarke’s $G_2$-instantons converge outside the associative $S^3$ to $A^{\lim }$. The fact that $A^{\lim }$ smoothly extends over $S^3$ can then be interpreted as a removable singularity phenomenon.

To state the result we now introduce some notation for the re-scaling we wish to perform: for $p \in S^3$ and $\delta >0$ we define the map $s^p_{\delta }$ from the unit ball $B_1\subseteq \mathbb {R}^4$ by

$$\begin{aligned} s^p_{\delta } : B_1\subseteq \mathbb {R}^4 \rightarrow B_{\delta }\times \{p\}\subseteq \mathbb {R}^4\times S^3,\quad x \mapsto (\delta x,p). \end{aligned}$$

Recall that if we view $\mathbb {R}^4{\setminus }\{0\}=\mathbb {R}^+_t\times S^3$ then the basic ASD instanton on $\mathbb {R}^4$ with scale $\lambda >0$ can be written as

3.16 $$\begin{aligned} A^{\text {ASD}}_{\lambda }= \frac{\lambda t^2}{1+\lambda t^2} \sum _{i=1}^3T_i\otimes \eta _i^+ . \end{aligned}$$

##### Theorem 6

Let $\lbrace A^{x_1} \rbrace$ be a sequence of Clarke’s $G_2$-instantons from Theorem 4 with $x_1 \rightarrow + \infty$.

- Given any $\lambda >0$, there is a sequence of positive real numbers $\delta =\delta (x_1,\lambda ) \rightarrow 0$ as $x_{1} \rightarrow + \infty$ such that: for all $p \in S^3$, $(s^p_{\delta })^* A^{x_1}$ converges uniformly with all derivatives to the basic ASD instanton $A^{\text {ASD }}_{\lambda }$ on $B_1\subseteq \mathbb {R}^4$ as in (3.16).
- The connections $A^{x_1}$ converge uniformly with all derivatives to $A^{\lim }$ given in Theorem 5 on every compact subset of $(\mathbb {R}^4 {\setminus } \{0\})\times S^3$ as $x_1\rightarrow +\infty$.

##### Proof

We prove the two parts independently.

- We view the basic instanton $A^{\text {ASD}}_{\lambda }$ in (3.16) as defined on $\mathbb {R}^4\times \{p\}$. Using the formula for $A^{x_1}$ in Theorem 4 and the expansions of $A_1$ and $B_1$ near 0 in Example 1 from Appendix A, we compute, for $t< 1$,
  $$\begin{aligned} \left( s^p_{\delta }\right) ^* A^{x_1}= & {} A_1(\delta t ) x(\delta t ) T_i\otimes \eta _i^+ = \frac{2x_1 A_1^2(\delta t)}{1+x_1 \left( B_1^2(\delta t) - \frac{1}{3} \right) } T_i\otimes \eta _i^+ \\= & {} \frac{x_1\delta ^2 t^2/2 + O(x_1 \delta ^4 t^4)}{1+x_1\delta ^2 t^2/2 + O(x_1 \delta ^4 t^4)} T_i \otimes \eta _i^+ . \end{aligned}$$
  Hence, setting $\delta = \delta (x_1,\lambda )= \sqrt{2 \lambda / x_1}$ we have that for every $k \in \mathbb {N}_0$, there is $c_k>0$, independent of $\lambda$ and $x_1$, such that
  $$\begin{aligned} \left\| \left( s^p_{\delta }\right) ^* A^{x_1} - A^{\text {ASD}}_{\lambda } \right\| _{C^k(B_1)} \le c_k \frac{\lambda ^2}{x_1}. \end{aligned}$$
  Therefore, given $\epsilon >0$, we have for any $x_1\ge c_k \lambda ^2/ \epsilon$ that
  $$\begin{aligned} \left\| \left( s^p_{\delta }\right) ^* A^{x_1} - A^{\text {ASD}}_{\lambda } \right\| _{C^k(B_1)} \le \epsilon , \end{aligned}$$
  demonstrating the claimed convergence.
- We take the explicit formulas for $A^{x_1}$ and $A^{\lim }$ in Theorems 4 and 5 and compute
  $$\begin{aligned} \vert A^{x_1} - A^{\lim } \vert= & {} \frac{A_1^2(t)}{\frac{1}{2}\big (B_1^2(t)-\frac{1}{3}\big ) } \ \left| \frac{x_1 \big (B_1^2(t)-\frac{1}{3}\big )}{1+x_1 \big (B_1^2(t)-\frac{1}{3}\big ) } -1 \right| \left| \sum _{i=1}^3T_i\otimes \eta _i^+ \right| \\\le & {} \frac{c A_1(t)}{\frac{1}{2}\big (B_1^2(t)-\frac{1}{3}\big ) } \frac{1}{1+x_1 \big (B_1^2(t)-\frac{1}{3}\big ) }, \end{aligned}$$
  for some constant $c>0$. Recall that in the coordinate $r\in [1,+\infty )$ from (2.22) we have $B_1(r)=r/\sqrt{3}$ by (2.23). Hence, $B_1^2-\frac{1}{3}$ is bounded and bounded away from zero on every compact $K\subseteq (\mathbb {R}^4 {\setminus } \{0\})\times S^3$. Thus, for every such *K* there is some (possibly other) constant $c>0$ such that
  3.17 $$\begin{aligned} \vert A^{x_1} - A^{\lim } \vert \le \frac{c}{1+x_1}, \end{aligned}$$
  and we have similar estimates for the derivatives of $A^{x_1}-A^{\lim }$. By letting $x_1 \rightarrow + \infty$ the right-hand side of (3.17) tends to zero as required.

$$\square $$

##### Remark 7

As already mentioned, the fact that $A^{\lim }$ smoothly extends over $S^3$ is an example of a removable singularity phenomenon. It follows from Tian and Tao’s work [23, 24] that such phenomena occur more generally provided that the $G_2$-instanton is invariant under a group action all of whose orbits have dimension greater than or equal to 3 (codimension less than or equal to 4).

Even though the $G_2$-instantons $A^{x_1}$ and $A_{\lim }$ do not have finite energy, and so the results of [23] do not immediately apply, we now show that we do have the expected energy concentration along the associative $S^3$. Below, we let $\delta _{\lbrace 0 \rbrace \times S^3}$ denote the delta current associated with $\lbrace 0 \rbrace \times S^3$.

##### Corollary 2

The function $\vert F_{A^{x_1}} \vert ^2 - \vert F_{A_{\lim }} \vert ^2$ is integrable for all $x_1>0$. Moreover, as $x_1 \rightarrow + \infty$ it converges to $8 \pi ^2 \delta _{\lbrace 0 \rbrace \times S^3}$ as a current, i.e. for all compactly supported functions *f* we have

$$\begin{aligned} \lim _{x_1 \rightarrow + \infty }\int _{\mathbb {R}^4 \times S^3} f \left( \vert F_{A^{x_1}} \vert ^2 - \vert F_{A_{\lim }} \vert ^2\right) \ {{\mathrm{dvol}}}_g = 8 \pi ^2 \int _{\lbrace 0 \rbrace \times S^3} f \ {{\mathrm{dvol}}}_{g \vert _{\lbrace 0 \rbrace \times S^3}}. \end{aligned}$$

##### Proof

First, a computation using (3.12) shows that

3.18 $$\begin{aligned} \vert F_{A^{x_1}} \vert ^2 - \vert F_{A_{\lim }} \vert ^2 = \sum _{n=0}^3 \sum _{k=0}^{10} \frac{ 6q_{n,k} (r-1)^k x_1^n }{(r+1)^4 r^6 (r^2 x_1 -x_1 +3)^4} , \end{aligned}$$

for some (explicit) $q_{n,k} \in \mathbb {R}$. The claimed integrability of $\vert F_{A^{x_1}} \vert ^2 - \vert F_{A_{\lim }} \vert ^2$ now follows. Moreover, for future reference we mention here that

3.19 $$\begin{aligned} q_{3,0}=0=q_{3,1}, \quad \text {and} \quad q_{2,0}=2529. \end{aligned}$$

Next we prove the claim convergence of $\vert F_{A^{x_1}} \vert ^2 - \vert F_{A_{\lim }} \vert ^2$ by showing that

3.20 $$\begin{aligned} \lim _{x_1 \rightarrow + \infty } \int _K \left( \vert F_{A^{x_1}} \vert ^2 - \vert F_{A_{\lim }} \vert ^2\right) {{\mathrm{dvol}}}_g \end{aligned}$$

vanishes if $K \subset (\mathbb {R}^4 \backslash \lbrace 0 \rbrace ) \times S^3$ is compact and equals $8 \pi ^2 \ Vol( \lbrace 0 \rbrace \times S^3)$ if $\lbrace 0 \rbrace \times S^3 \subset K$. Notice that $\vert F_{A^{x_1}} \vert ^2 - \vert F_{A_{\lim }} \vert ^2$ is $\mathrm{SU}(2)^3$-invariant and so it is enough to consider its integral over $\mathrm{SU}(2)^3$-invariant subsets *K* of $\mathbb {R}^4 \times S^3$. First we consider the case when *K* is a compact subset of $(\mathbb {R}^4 \backslash \lbrace 0 \rbrace ) \times S^3$. Then, Theorem 6(b) guarantees that $\vert F_{A^{x_1}} \vert ^2 - \vert F_{A_{\lim }} \vert ^2$ converges uniformly to 0 in *K* and so (3.20) is zero by the dominated convergence theorem.

To examine the case where $\lbrace 0 \rbrace \times S^3 \subset K$ we first show that, as currents, we have

3.21 $$\begin{aligned} \lim _{x_1 \rightarrow + \infty } (\vert F_{A^{x_1}} \vert ^2 - \vert F_{A_{\lim }} \vert ^2 ) = \lim _{x_1 \rightarrow + \infty } \frac{ 6 \cdot 2592 x_1^2 }{(r+1)^4 r^6 (r^2 x_1 -x_1 +3)^4}. \end{aligned}$$

Recall from Sect. 2.2.1 that we can identify $(\mathbb {R}^4{\setminus }\{0\})\times S^3\cong (1,\infty )\times S^3\times S^3$, with *r* the coordinate on $(1,\infty )$. For $f \in C^{\infty }_c(\mathbb {R}^4 \times S^3 , \mathbb {R})$ we can then compute that

3.22 $$\begin{aligned}&\left| \int _{\mathbb {R}^4 \times S^3} f \frac{(r-1)^k x_1^n}{(r+1)^4 r^6 (r^2 x_1 -x_1 +3)^4} {{\mathrm{dvol}}}_g \right| \nonumber \\&\quad \le \Vert f \Vert _{L^{\infty }} \int _{1}^{+\infty } \frac{(r-1)^k x_1^n}{(r+1)^4 r^6 (r^2 x_1 -x_1 +3)^4} \frac{r^6(1-r^{-3}) Vol(\mathbb {S}^3_1)^2}{3^4 \sqrt{3}} dr , \end{aligned}$$

where $Vol(\mathbb {S}^3_1)=2\pi ^2$ denotes the volume of the unit 3-sphere in $\mathbb {R}^4$. Let $I_{n,k}(x_1)$ denote the integral on the right-hand side of (3.22) and let $\epsilon >0$. To examine $I_{n,k}(x_1)$, we separate it into two integrals: one over $[1,1+\epsilon ]$ and the other over $[1+\epsilon , + \infty )$. The second of these integrals can be easily seen to be finite and of order $x_1^{n-4}$ (independently of *k*), hence it vanishes as $x_1 \rightarrow + \infty$ since $n\le 3$. The first integral over $[1,1+\epsilon ]$, and thus $I_{n,k}(x_1)$ by the preceding argument, can be bounded as follows for some constant *c*:

3.23 $$\begin{aligned} I_{n,k}(x_1) \le c x_1^{n-4} \int _1^{1+\epsilon } \frac{(r-1)^{k+1}}{(r- \sqrt{1-3/x_1})^4} dr + O(x_1^{n-4}). \end{aligned}$$

The integral on the right-hand side of (3.23) can now be computed to be of order $O(x_1^{2-k})$ for $k=0,1$, $O(\log (x_1))$ for $k=2$ and *O*(1) for $k>2$. Letting $x_1 \rightarrow + \infty$ we see that (3.23) vanishes unless $k=0$ and $n=2,3$, or $k=1$ and $n=3$. From (3.19) we then see that (3.21) holds as desired.

Now suppose $\lbrace 0 \rbrace \times S^3 \subset K$. Rewriting (3.20) we have from the first part of the proof that

$$\begin{aligned}&\lim _{x_1 \rightarrow + \infty } \int _K \left( \vert F_{A^{x_1}} \vert ^2 - \vert F_{A_{\lim }} \vert ^2\right) {{\mathrm{dvol}}}_g \\&\quad = \lim _{x_1 \rightarrow + \infty } \int _{K \cap B_{\epsilon }(0 \times S^3)} \left( \vert F_{A^{x_1}} \vert ^2 - \vert F_{A_{\lim }} \vert ^2\right) {{\mathrm{dvol}}}_g. \end{aligned}$$

Hence, using (3.21) to compute the integral gives

$$\begin{aligned}&\lim _{x_1 \rightarrow + \infty }\int _{K} \frac{ 6 \cdot 2592 x_1^2 }{(r+1)^4 r^6 (r^2 x_1 -x_1 +3)^4} {{\mathrm{dvol}}}_g \\&\quad = \lim _{x_1 \rightarrow + \infty } \int _1^{1+\epsilon } \frac{ 6 \cdot 2592 x_1^2 (1-r^{-3}) }{(r+1)^4 r^6 (r^2 x_1 -x_1 +3)^4} \frac{2^3 Vol\left( \mathbb {S}^3_1\right) ^2}{3^4 \sqrt{3}} dr \\&\quad = \frac{4}{3\sqrt{3}} Vol\left( \mathbb {S}_1^3\right) ^2 = \frac{8 \pi ^2}{3 \sqrt{3}} Vol\left( \mathbb {S}_1^3\right) . \end{aligned}$$

Now recall that the metric at the zero section is $\left( \frac{2}{\sqrt{3}} \right) ^2 (\eta _1^- \otimes \eta _1^- + \eta _2^- \otimes \eta _2^- + \eta _3^- \otimes \eta _3^-)$, hence its volume form is $1 / 3\sqrt{3}$ times that of $\mathbb {S}^3_1$. The result then follows. $\square$

##### Remark 8

The sequence of instantons with curvature concentrating along the associative $S^3$ determines a Fueter section, as in [9, 14, 27], from $S^3$ to the bundle of moduli spaces of anti-self-dual connections associated to the normal bundle. The section thus determined is constant, taking value at the basic instanton on $\mathbb {R}^4$. The Yang–Mills energy of the basic instanton is $8\pi ^2$, so Corollary 2 confirms the expected “conservation of energy” formula (c.f. [23]).

## $\mathrm{SU}(2)^2\times U(1)$-invariant $G_2$-instantons

The main goal of this section is to investigate SU(2)$^2$-invariant $G_2$-instantons on the Brandhuber et al. (BGGG) $G_2$-manifold $\mathbb {R}^4\times S^3$ from Sect. 2.2.2. We will restrict ourselves to instantons that enjoy an extra *U*(1)-symmetry present in the underlying geometry. As already mentioned, all of the known complete SU(2)$^2$-invariant $G_2$-manifolds of cohomogeneity-1 enjoy an extra *U*(1)-symmetry and so the analysis of $\mathrm{SU}(2)^2\times U(1)$-invariant $G_2$-instantons provides a natural stepping stone to a complete understanding of SU(2)$^2$-invariant $G_2$-instantons.

We begin in Sect. 4.1 by deriving the ODEs determining $G_2$-instantons in this setting by simplifying the general ODEs and constraint in Lemma 3. We then determine the necessary conditions ensuring that solutions to these ODEs smoothly extend across the singular orbit in the Bryant–Salamon (BS), BGGG and Bogoyavlenskaya $G_2$-manifolds in Sect. 4.2. In the final section Sect. 4.3, we explicitly describe the $G_2$-instantons which exist near the singular orbit. This leads to a stronger classification result in the BS case, and existence and non-existence results for global $G_2$-instantons in the BGGG case.

### The $\mathrm{SU}(2)^2\times U(1)$-invariant ODEs

We shall now rewrite the ODEs from Lemma 3 in this $\mathrm{SU}(2)^2\times U(1)$-invariant setting. As for the $\mathrm{SU}(2)^3$-invariant case it will be convenient to rescale the fields $a_i^{\pm }$, for $i=1,2,3$, defining the connection 1-form as in (2.27). We thus define

$$\begin{aligned} c^+_i= \frac{a^+_i}{A_i}, \quad c^-_i = \frac{a^-_i}{B_i} \end{aligned}$$

so that the connection 1-form is

$$\begin{aligned} A=\sum _{i=1}^3 A_ic^+_i\otimes \eta _i^++B_ic^-_i\otimes \eta _i^-. \end{aligned}$$

In these terms we can use (2.16)–(2.19) to obtain the general SU(2)$^2\times U(1)$-invariant $G_2$-instanton equations for *A* from Lemma 3 as follows:

4.1 $$\begin{aligned} \dot{c}_1^+ + \frac{1}{2}\left( \frac{A_1}{B_2^2}-\frac{A_1}{A_2^2}\right) c_1^+&= \frac{1}{2} \left[ c_2^-, c_3^-\right] -\frac{1}{2} \left[ c_2^+, c_3^+\right] , \end{aligned}$$

4.2 $$\begin{aligned} \dot{c}_2^+ +\frac{1}{2}\left( \frac{A_2^2+B_1^2+B_2^2}{A_2B_1B_2} -\frac{A_1^2+2A_2^2}{A_1A_2^2}\right) c_2^+&=\frac{1}{2} \left[ c_3^- , c_1^-\right] -\frac{1}{2} \left[ c_3^+ , c_1^+\right] , \end{aligned}$$

4.3 $$\begin{aligned} \dot{c}_3^+ +\frac{1}{2}\left( \frac{A_2^2+B_1^2+B_2^2}{A_2B_1B_2} -\frac{A_1^2+2A_2^2}{A_1A_2^2}\right) c_3^+&= \frac{1}{2} \left[ c_1^- , c_2^-\right] -\frac{1}{2} \left[ c_1^+ , c_2^+\right] ,\end{aligned}$$

4.4 $$\begin{aligned} \dot{c}_1^- + \left( \frac{A_2^2+B_1^2+B_2^2}{A_2B_1B_2}\right) c_1^-&= \frac{1}{2} \left[ c_2^-, c_3^+\right] +\frac{1}{2}\left[ c_2^+, c_3^-\right] , \end{aligned}$$

4.5 $$\begin{aligned} \dot{c}_2^- + \frac{1}{2}\left( \frac{A_2^2+B_1^2+B_2^2}{A_2B_1B_2} +\frac{A_1^2+2B_2^2}{A_1B_2^2}\right) c_2^-&= \frac{1}{2} \left[ c_3^- , c_1^+\right] +\frac{1}{2} \left[ c_3^+ , c_1^-\right] , \end{aligned}$$

4.6 $$\begin{aligned} \dot{c}_3^- + \frac{1}{2}\left( \frac{A_2^2+B_1^2+B_2^2}{A_2B_1B_2} +\frac{A_1^2+2B_2^2}{A_1B_2^2}\right) c_3^-&= \frac{1}{2} \left[ c_1^- , c_2^+\right] +\frac{1}{2} \left[ c_1^+ , c_2^-\right] , \end{aligned}$$

together with the constraint

4.7 $$\begin{aligned} \sum _{i=1}^3 \left[ c_i^+ , c_i^-\right] =0. \end{aligned}$$

We now wish to simplify these equations further using an additional *U*(1)-symmetry in the ambient geometry. This extra symmetry in the known complete SU(2)$^2$-invariant cohomogeneity-1 $G_2$-manifolds from Sect. 2.2 can be encoded, for example, by regarding $S^3 \times S^3$ as $\mathrm{SU}(2)^2 \times U(1) / \Delta U(1)$, with $\Delta U(1)$ acting via

$$\begin{aligned} e^{i \theta } \cdot (g_1,g_2,e^{i \alpha })= (g_1 {{\mathrm{diag}}}(e^{i \theta }, e^{-i \theta }) , g_2 {{\mathrm{diag}}}(e^{i \theta }, e^{-i \theta }) , e^{i (\alpha + \theta )}). \end{aligned}$$

With this in hand, we can derive the simplified ODEs in this setting.

#### Proposition 6

Let *A* be an $\mathrm{SU}(2)^2 \times U(1)$-invariant $G_2$-instanton on $\mathbb {R}^+\times \mathrm{SU}(2)^2\cong \mathbb {R}^+\times (\mathrm{SU}(2)^2\times U(1)/\Delta U(1))$ with gauge group $\mathrm{SU}(2)$. There is a standard basis $\lbrace T_i \rbrace _{i=1}^3$ of $\mathfrak {su}(2)$, i.e. with $[T_i,T_j]=2\epsilon _{ijk} T_k$, such that (up to an invariant gauge transformation) we can write

4.8 $$\begin{aligned} A= & {} A_1 f^{+} T_1\otimes \eta _1^{+} + A_2g^{+} \left( T_2\otimes \eta _2^{+} + T_3\otimes \eta _3^{+} \right) \nonumber \\&+ B_1 f^{-} T_1\otimes \eta _1^{-} + B_2 g^{-} \left( T_2\otimes \eta _2^{-} + T_3\otimes \eta _3^{-}\right) , \end{aligned}$$

with $$f^{\pm }$$, $$g^{\pm }: \mathbb {R}^+ \rightarrow \mathbb {R}$$ satisfying

4.9 $$\begin{aligned} \dot{f}^++\frac{1}{2}\left( \frac{A_1}{B_2^2}-\frac{A_1}{A_2^2}\right) f^+&= (g^-)^2 - (g^+)^2, \end{aligned}$$

4.10 $$\begin{aligned} \dot{g}^+ +\frac{1}{2}\left( \frac{A_2^2+B_1^2+B_2^2}{A_2B_1B_2} -\frac{A_1^2+2A_2^2}{A_1A_2^2}\right) g^+&= f^-g^- - f^+ g^+ ,\end{aligned}$$

4.11 $$\begin{aligned} \dot{f}^- + \left( \frac{A_2^2+B_1^2+B_2^2}{A_2B_1B_2}\right) f^-&= 2g^- g^+, \end{aligned}$$

4.12 $$\begin{aligned} \dot{g}^- + \frac{1}{2}\left( \frac{A_2^2+B_1^2+B_2^2}{A_2B_1B_2} +\frac{A_1^2+2B_2^2}{A_1B_2^2}\right) g^-&= g^- f^+ + g^+ f^- . \end{aligned}$$

#### Proof

We must consider $\mathrm{SU}(2)^2 \times U(1)$-homogeneous $\mathrm{SU}(2)$-bundles over $S^3 \times S^3\cong \mathrm{SU}(2)^2 \times U(1) / \Delta U(1)$. Such bundles are parametrized by isotropy homomorphisms $\lambda : \Delta U(1) \rightarrow \mathrm{SU}(2)$, which take the form $\lambda _k(e^{i \theta })= {{\mathrm{diag}}}(e^{ik \theta }, e^{-i k \theta })$. We take the complement of the isotropy algebra $\Delta \mathfrak {u}(1)$ to be $\mathfrak {m}= \mathfrak {su}^+(2) \oplus \mathfrak {su}^-(2) \oplus 0$. The canonical invariant connection on the bundle

$$\begin{aligned} P_k = (\mathrm{SU}(2)^2 \times U(1) ) \times _{(\Delta U(1), \lambda _k)} \mathrm{SU}(2) \end{aligned}$$

is given by $d \lambda _k = T_1\otimes k d \theta$, where the $\lbrace T_i \rbrace _{i=1}^3$ form a standard basis for $\mathfrak {su}(2)$ and $\theta$ is the periodic coordinate on *U*(1). Wang’s theorem [28] states that any other invariant connection *a* on $P_k$ can be written as $d \lambda _k + \Lambda _k$, where $\Lambda _k$ is the left-invariant extension to $\mathrm{SU}(2)^2 \times U(1)$ of a morphism of $\Delta U(1)$-representations $\Lambda _k : (\mathfrak {m} , {{\mathrm{Ad}}}) \rightarrow (\mathfrak {su}(2) , {{\mathrm{Ad}}}\circ \lambda _k)$. Splitting into irreducibles, we have

$$\begin{aligned} \mathfrak {m} = (\mathbb {R} \oplus \mathbb {C}_2) \oplus ( \mathbb {R} \oplus \mathbb {C}_2) \oplus 0, \end{aligned}$$

while $(\mathfrak {su}(2) , {{\mathrm{Ad}}}\circ \lambda _k)$ splits as $\mathbb {R}\oplus \mathbb {C}_{2k}$. Therefore, other invariant connections exist only when $k=1$, in which case we can apply a gauge transformation so that

$$\begin{aligned} a= & {} T_1\otimes d \theta + A_1 f^{+} T_1\otimes \eta _1^{+} + A_2 g^{+} \left( T_2\otimes \eta _2^{+} + T_3\otimes \eta _3^{+} \right) \\&+B_1 f^{-} T_1\otimes \eta _1^{-} + B_2 g^{-} \left( T_2\otimes \eta _2^{-} + T_3\otimes \eta _3^{-} \right) , \end{aligned}$$

where $f^{\pm }$, $g^{\pm }$ are constants. We now pull this back to SU(2)$^2$ via the inclusion map $\mathrm{SU}(2)^2 \rightarrow \mathrm{SU}(2)^2 \times U(1)$ and extend it to $\mathbb {R}^+\times \mathrm{SU}(2)^2$ to obtain

$$\begin{aligned} A= & {} \gamma \big (A_1 f^{+} T_1\otimes \eta _1^{+} + A_2 g^{+} \big (T_2\otimes \eta _2^{+} + T_3\otimes \eta _3^{+} \big )\big )\gamma ^{-1} \\&+ \gamma \big ( B_1 f^{-} T_1\otimes \eta _1^{-} + B_2 g^{-} \big (T_2\otimes \eta _2^{-} + T_3\otimes \eta _3^{-} \big ) \big )\gamma ^{-1} , \end{aligned}$$

for functions $\gamma :\mathbb {R}^+\rightarrow \mathrm{SU}(2)$ and $f^{\pm },g^{\pm }:\mathbb {R}^+\rightarrow \mathbb {R}$.

We now turn our attention to such connections *A* which can solve the $G_2$-instanton Eqs. (4.1)–(4.7). We first see that the constraint (4.7) is satisfied and we claim that the evolution equations imply the ODEs (4.9)–(4.12) and that $\dot{\gamma }=0$. Observe that (4.1) becomes

$$\begin{aligned} \dot{f}^+ T_1 + f^+ \left[ \gamma ^{-1}\dot{\gamma },T_1\right] + \frac{1}{2}\left( \frac{A_1}{B_2^2}-\frac{A_1}{A_2^2}\right) f^+ T_1 = ((g^{-})^2 - (g^{+})^2 ) T_1 . \end{aligned}$$

We conclude that $f^+[\gamma ^{-1}\dot{\gamma },T_1]=0$ and obtain (4.9). Entirely analogous computations yield

$$\begin{aligned} g^+\left[ \gamma ^{-1}\dot{\gamma },T_i\right] =0,\quad f^-\left[ \gamma ^{-1}\dot{\gamma },T_1\right] =0,\quad g^-\left[ \gamma ^{-1}\dot{\gamma },T_i\right] =0 \end{aligned}$$

for $i=2,3$, as well as (4.10)–(4.12). Hence, if $A\ne 0$ we obtain $\dot{\gamma }=0$ and so *A* takes the form (4.8) as required. $\square$

#### Remark 9

In the setup of the Proposition 6, we have

$$\begin{aligned} c_1^{\pm }= f^{\pm } T_1 , \ c_2^{\pm }=g^{\pm } T_2, \ c_3^{\pm }=g^{\pm } T_3, \end{aligned}$$

where $f^{\pm }, g^{\pm } : \mathbb {R}^+ \rightarrow \mathbb {R}$ satisfy the ODEs (4.9)–(4.12).

### Initial conditions

To investigate $\mathrm{SU}(2)^2 \times U(1)$-invariant $G_2$-instantons *A* on the BGGG $G_2$-manifold $\mathbb {R}^4\times S^3$, as well as the BS and Bogoyolavenskaya cases, we study the conditions for *A* to extend smoothly over the singular orbit $\mathrm{SU}(2)^2 / \Delta \mathrm{SU}(2) \cong \{0\}\times S^3$.

As in Sect. 3.2, we have two bundles $P_{\lambda }$ as in (3.5) to consider, where $\lambda : \Delta \mathrm{SU}(2) \rightarrow \mathrm{SU}(2)$ is either trivial $\lambda =1$ or the identity $\lambda ={{\mathrm{id}}}$. Recall from Proposition 6 that *A* takes the form in (4.8), determined by functions $f^{\pm },g^{\pm }:\mathbb {R}_{\ge 0}\rightarrow \mathbb {R}$. The next result gives the conditions on $f^{\pm }, g^{\pm }$ so that such *A* extends over a singular orbit at $t=0$. To state it, we observe that Lemma 8 in Appendix A shows that for any $\mathrm{SU}(2)^2 \times U(1)$-invariant $G_2$-metric which smoothly extends over a singular orbit at $t=0$ must be of the form (2.13) for functions $A_1$, $A_2=A_3$, $B_1$, $B_2=B_3$ which admit Taylor expansions of the form

4.13 $$\begin{aligned} A_i(t)=\frac{t}{2} + t^3 C_i(t)\quad \text {and}\quad B_i(t) = b + t^2 D_i(t), \end{aligned}$$

for some $b \in \mathbb {R}{\setminus }\{0\}$ and real analytic even functions $C_1$, $C_2=C_3$, $D_1$, $D_2=D_3$ with $D_1(0)=D_2(0)$. The explicit values of $C_1(0)$, $C_2(0)$ and $D_1(0)$ for the BS and BGGG cases can be found in Examples 1–2 in Appendix A.

#### Lemma 5

The connection *A* in (4.8) extends smoothly over the singular orbit $S^3$ if and only if $f^+$ and $g^+$ are odd, $f^-$ and $g^-$ are even, and, using the notation in (4.13), their Taylor expansions around $t=0$ are:

- either
  $$\begin{aligned} f^{-}&=f_2^- t^2 + O(t^4),\quad g^-= g_2^- t^2 + O(t^4),\\ f^{+}&= f_1^+ t + O(t^3),\quad g^+= g_1^+ t + O(t^3), \end{aligned}$$
  in which case *A* extends smoothly as a connection on $P_{1}$;
- or
  $$\begin{aligned} f^{-}&= b_0^- + b_2^- t^2 + O(t^4) ,\quad g^-= b_0^- + b_2^- t^2 + O(t^4), \\ f^{+}&= \frac{2}{t} + \left( b_2^+-4C_1(0) \right) t + O(t^3) ,\quad g^+= \frac{2}{t} + \left( b_2^+-4C_2(0)\right) t + O(t^3), \end{aligned}$$
  in which case *A* extends smoothly as a connection on $P_{{{\mathrm{id}}}}$.

#### Proof

We start with $P_{{{\mathrm{id}}}}$, i.e. where the homomorphism $\lambda ={{\mathrm{id}}}$, as it is slightly more involved. The canonical invariant connection on $P_{{{\mathrm{id}}}} \rightarrow S^3$ is $A^{\text {can}}$ in (3.6). We must then apply Lemma 10 from Appendix A to the $\mathfrak {su}(2)$-valued 1-form

$$\begin{aligned} A-A^{\text {can}}= & {} \left( A_1 f^{+}-1\right) T_1\otimes \eta _1^{+} + \left( A_2 g^+ -1\right) \left( T_2\otimes \eta _2^{+} + T_3\otimes \eta _3^{+} \right) \\&+ B_1 f^{-} T_1\otimes \eta _1^{-} + B_2 g^- \left( T_2\otimes \eta _2^{-} + T_3\otimes \eta _3^{-}\right) . \end{aligned}$$

We deduce that $A_1 f^{+}-1$, $A_2 g^+ -1$, $B_1 f^{-}$ and $B_2 g^-$ are all even. Moreover, the first two of these must admit Taylor expansions of the form $\frac{b_2^+}{2} t^2 + O(t^4)$, for some $b_2^+ \in \mathbb {R}$, while the last two have expansions of the form $\frac{b_0^-}{2} + \frac{b_2^-}{2} t^2 + O(t^4)$, for some $b_0^{-}$, $b_2^{-} \in \mathbb {R}$. Using (4.13), we deduce that

$$\begin{aligned} f^{+}= \frac{2}{t} + \left( b_2^+ -4C_1(0) \right) t + O(t^3), \quad g^+= \frac{2}{t} + \left( b_2^+ -4C_2(0)\right) t + O(t^3), \end{aligned}$$

and, since $D_1(0)=D_2(0)$ and $b\ne 0$, we have $f^-=h^-+O(t^4)$, $g^-=h^-+O(t^4)$ where

$$\begin{aligned} h^-=\frac{b_0^-}{2b} + \left( \frac{b_2^-}{2b} -\frac{b_0^-}{2b^2} D_1(0) \right) t^2. \end{aligned}$$

The statement for $P_{{{\mathrm{id}}}}$ then follows.

We now turn to $P_1$. Here we instead apply Lemma 10 to the 1-form *A* itself and conclude that $A_1 f^{+}$, $A_2 g^+$, $B_1 f^{-}$, $B_2 g^-$ must all be even and vanish at $t=0$. Hence, by (4.13), we see that $f^+,g^+$ are odd and $f^-,g^-$ are even such that$f^-(0)=g^-(0)=0$. $\square$

### Solutions

We now investigate existence of solutions of the $\mathrm{SU}(2)^2\times U(1)$-invariant $G_2$-instanton equations with gauge group $\mathrm{SU}(2)$ on the BS, BGGG and Bogoyavlenskaya $G_2$-manifolds $\mathbb {R}^4\times S^3$. There are two cases: when the bundle is $P_1$ or $P_{{{\mathrm{id}}}}$, in the notation of the previous subsection. In both cases we explicitly classify the invariant $G_2$-instantons defined near the singular orbit which extend smoothly and, as a consequence, extend our uniqueness result for $G_2$-instantons on the BS $\mathbb {R}^4\times S^3$ to the case of $\mathrm{SU}(2)^2 \times U(1)$-symmetry, and obtain both *existence* and *non-existence* results for $G_2$-instantons with decaying curvature on the BGGG $\mathbb {R}^4\times S^3$.

#### Solutions smoothly extending on $P_{1}$

We shall now investigate the existence of solutions that smoothly extend over the singular orbit $S^3 = \mathrm{SU}(2)^2/\Delta \mathrm{SU}(2)$ on the bundle $P_1$. The main results are Proposition 7 and Theorems 7–9. Proposition 7 shows the existence of a 2-parameter family of $G_2$-instantons in a neighbourhood of the singular orbit, so there is at most a 2-parameter family of $\mathrm{SU}(2)^2\times U(1)$-invariant $G_2$-instantons on $P_1$ on the BS, BGGG and Bogoyavlenskaya $G_2$-manifolds. Theorem 7 shows that in the BS case, just a 1-parameter family of these local instantons extends, and these are either given by Clarke’s $\mathrm{SU}(2)^3$-invariant examples from Theorem 4 or are abelian. Theorems 8 and 9 show that, unlike the BS case, there is a 2-parameter family of local $G_2$-instantons which extend to the whole BGGG $\mathbb {R}^4\times S^3$ so that their curvature is bounded, as well as a 2-parameter family which do not extend so as to have bounded curvature.

##### Proposition 7

Let $X \subset \mathbb {R}^4\times S^3$ contain the singular orbit $\{0\}\times S^3$ of the $\mathrm{SU}(2)^2 \times U(1)$ action and be equipped with an $\mathrm{SU}(2)^2 \times U(1)$-invariant holonomy $G_2$-metric. There is a 2-parameter family of $\mathrm{SU}(2)^2 \times U(1)$-invariant $G_2$-instantons *A* with gauge group $\mathrm{SU}(2)$ in a neighbourhood of the singular orbit in *X* smoothly extending over $P_{1}$.

Moreover, in the notation of Proposition 6 and (4.13), any such $G_2$-instanton *A* can be written as in (4.8) with $f^-=0=g^-$ and with $f^+$, $g^+$ solving the ODEs:

4.14 $$\begin{aligned} \dot{f}^+ + \frac{1}{2}\left( \frac{A_1}{B_2^2}-\frac{A_1}{A_2^2}\right) f^+= & {} - (g^+)^2, \end{aligned}$$

4.15 $$\begin{aligned} \dot{g}^+ + \frac{1}{2}\left( \frac{A_2^2+B_1^2+B_2^2}{A_2B_1B_2} -\frac{A_1^2+2A_2^2}{A_1A_2^2}\right) g^+= & {} - f^+ g^+ , \end{aligned}$$

subject to $f^+(t)=f_1^+ t+ t^3 u_1(t)$, $g^+(t)=g_1^+ t+ t^3 u_2(t)$, where $f_1^+,g_1^+ \in \mathbb {R}$ and the $u_i$ are real analytic functions such that

4.16 $$\begin{aligned} u_1(0)= & {} -f_1^+\left( \frac{1}{8 b^2} + 2C_2(0)-C_1(0) \right) -\frac{(g^+_1)^2}{2} , \end{aligned}$$

4.17 $$\begin{aligned} u_2(0)= & {} -\frac{g_1^+}{2}\left( \frac{1}{4 b^2} + 2 C_1(0) + f_1^+ \right) . \end{aligned}$$

##### Proof

It is convenient to study our initial value problem by writing

$$\begin{aligned} f^+(t)&=f_1^+ t+ t^3 u_1(t),\quad g^+(t)=g_1^+ t+ t^3 u_2(t),\\ f^-(t)&=t^2 v_1(t),\quad g^{-}(t)= t^2 v_2(t), \end{aligned}$$

for some real analytic functions $u_1,u_2,v_1,v_2$, which we can do by Lemma 5. In this way the ODEs for $G_2$-instantons from Proposition 6 turn into ODEs for these 4 functions, which we write as $X(t)=(u_1(t),u_2(t), v_1(t), v_2(t))$. A lengthy but otherwise straightforward computation yields the regular singular initial value problem at $t=0$:

$$\begin{aligned} \frac{dX}{dt}= \frac{M_{-1}(X)}{t}+M(t,X), \end{aligned}$$

where *M*(*t*, *X*) is real analytic in the first coordinate and

$$\begin{aligned} M_{-1}(X)=\,\,&\Big ( -2 u_1 - \left( \frac{1}{4 b^2} + 4C_2(0)-2C_1(0) \right) f_1^+ - \big (g_1^+\big )^2 ,\\&-2u_2 - \left( \frac{1}{4 b^2} + 2C_1(0)+f_1^+ \right) g_1^+ , -6 v_1, -6 v_2 \Big ). \end{aligned}$$

The existence and uniqueness theorem for singular initial value problems ([18], see also Theorem 4.7 in [13] for a clearer statement) applies if and only if $M_{-1}(X(0))=0$ and $dM_{-1}(X(0))$ does not have any positive integer as an eigenvalue. Since $dM_{-1}(X(0))$ is diagonal with eigenvalues $-2,-2,-6,-6$, we only need $v_1(0)=0=v_2(0)$ and $u_1(0)$, $u_2(0)$ as in (4.16), (4.17) to apply the existence and uniqueness theorem: this determines the possible initial values *X*(0), which are therefore parametrized by $f_1^+ , g_1^+ \in \mathbb {R}$. We conclude that there is a local two-parameter family of $G_2$-instantons with $\mathrm{SU}(2)^2 \times U(1)$-symmetry as claimed.

Notice that all these $G_2$-instantons have $v_1(0)=0=v_2(0)$. Thus, setting the smaller singular initial value problem above with $f^-$ and $g^-$ both vanishing gives the same local existence and uniqueness result, and hence the uniqueness implies that in fact $f^-(t)$, $g^-(t)$ must vanish identically for any solution extending smoothly over the singular orbit. The resulting ODEs (4.14), (4.15) then follow from Proposition 6. $\square$

##### Remark 10

Recall that the BS, BGGG and Bogoyavlenskaya $G_2$-metrics all have SU(2)$^2 \times U(1)$-symmetry and so Proposition 7 yields $G_2$-instantons in these cases.

Our first result shows that the sign of $g_1^+$ determines the sign of $g^+$.

##### Lemma 6

Let $(f^+,g^+)$ solve (4.14), (4.15). The sign of $g^+$ does not change as long as $f^+$ does not blow up, and if $g^+(t_0)=0$ for some $t_0> 0$ or if $g_1^+=0$ then $g^+\equiv 0$.

##### Proof

Suppose, for a contradiction, that the sign of $g^+$ changes. Then there is $t_0> 0$ such that $g^+(t_0)=0$. The ODE (4.15) implies that $\dot{g}^+(t_0)=0$ and thus $g^+\equiv 0$ (as $g^+$ solves a linear first order ODE), giving our contradiction. The same argument using (4.15) yields the statement. $\square$

##### Remark 11

The ODEs (4.14), (4.15) are invariant under $g^+\mapsto -g^+$. We may therefore exchange $g^+$ with $-g^+$ and, by virtue of Lemma 6, assume that $g_1^+\ge 0$, and thus $g^+\ge 0$, if we wish.

We first focus on the BS $G_2$-manifold $\mathbb {R}^4\times S^3$. It follows from Proposition 7 that there is at most a 2-parameter family of $\mathrm{SU}(2)^2\times U(1)$-invariant $G_2$-instantons defined globally on the BS $G_2$-manifold. We have a 1-parameter family of such instantons (with more symmetry) from Theorem 4 and a 1-parameter family of abelian examples from Corollary 1. We now show that these examples provide a complete classification.

##### Theorem 7

Let *A* be a $\mathrm{SU}(2)^2\times U(1)$-invariant $G_2$-instanton with gauge group $\mathrm{SU}(2)$ on the BS $G_2$-manifold $\mathbb {R}^4\times S^3$ which extends smoothly on $P_1$. Either *A* is $\mathrm{SU}(2)^3$-invariant, and so is given in Theorem 4; or it is reducible, in which case it has gauge group *U*(1) and is given in Corollary 1(a) with $x_2=x_3=0$.

##### Proof

In the BS case, using (3.1), we see that (4.14), (4.15) are now

4.18 $$\begin{aligned} \dot{f}^+-\frac{\dot{A}_1}{A_1}f^+=-(g^+)^2\quad \text {and}\quad \dot{g}^+-\frac{\dot{A}_1}{A_1}g^+=-f^+g^+. \end{aligned}$$

Let $F=f^+/A_1$ and $G=g^+/A_1$, and define $s\in [0,\infty )$ by $\frac{ds}{dt}=A_1$. If we let $f'=\frac{df}{ds}$ then (4.18) is equivalent to

4.19 $$\begin{aligned} F'=-G^2\quad \text {and}\quad G'=-FG. \end{aligned}$$

It follows from (4.19) that $F^2-G^2=c\in \mathbb {R}$, so we need only consider the ODE

4.20 $$\begin{aligned} F'=c-F^2. \end{aligned}$$

If $c<0$, the solutions to (4.20) satisfy $F^2(s)=-c\tan ^2(a-\sqrt{-c} s)$, which can then only exist for finite *s* and thus finite *t*.

If $c>0$ there are two types of solutions to (4.20): either $F^2(s)=c\tanh ^2(a+\sqrt{c}s)$ or $F^2=c$. The first solutions have $F^2-c<0$ which contradicts $F^2-c=G^2$. The second solutions force $G\equiv 0$, which give abelian instantons as in Corollary 1.

If $c=0$, then $F^2=G^2$, which means $F=\pm G$ so $f^+=\pm g^+$. By Remark 11, we may assume that $f^+=g^+$. In this case, *A* is SU(2)$^3$-invariant and the result then follows from Theorem 4. $\square$

We now focus attention on the BGGG $G_2$-manifold, though some of our results hold for the 1-parameter family of Bogoyavlenskaya metrics which includes the BGGG metric. It is natural in the study of $G_2$-instantons on non-compact $G_2$-manifolds to assume a decay condition on the curvature of the connection at infinity. The weakest reasonable assumption we can make is the curvature is bounded. In this setting we can prove both existence and non-existence results.

We first observe the conditions imposed on $f^+,g^+$ for the Bogoyavlensakaya metrics when the curvature is bounded.

##### Lemma 7

Let *A* be the $G_2$-instanton on one of the Bogoyavlenskaya $G_2$-manifolds induced by the pair $(f^+,g^+)$ as in Proposition 7. Then $|F_A|$ is bounded only if $g^+$ is bounded, and if both $f^+$ and $g^+$ are bounded then $|F_A|$ is bounded.

##### Proof

The $G_2$-instanton *A* induced by the pair $(f^+,g^+)$ has connection form as in (4.8) with $f^-=g^-=0$. Since $A=a(t)$, the curvature $F_A = dt \wedge \dot{a} + F_a$ of *A* can be computed from Lemma 2. Notice that $\vert F_A \vert ^2 = \vert \dot{a} \vert ^2 + \vert F_a \vert ^2$. Computing each of these terms separately we have

$$\begin{aligned} \vert F_a \vert ^2&= \frac{1}{4} \left( (g^+)^2 - \frac{A_1}{A_2^2} f^+ \right) ^2 + \frac{(g^+)^2}{2} \left( f^+-\frac{1}{A_1} \right) ^2 + \frac{A_1^2 (f^+)^2}{4 B_2^4} + \frac{A_2^2 (g^+)^2}{2 B_1^2 B_2^2},\\ \vert \dot{a} \vert ^2&= \frac{1}{4} \left( (g^+)^2-\frac{A_1 f^+}{A_2^2}+ \frac{A_1 f^+}{B_2^2} \right) ^2 + \frac{(g^+)^2}{2} \left( f^+-\frac{1}{A_1} + \frac{A_2}{B_1 B_2} \right) ^2, \end{aligned}$$

where in the second case we have used the $G_2$-instanton Eqs. (4.14), (4.15).

It follows from the work in [3] that, up to rescaling,

4.21 $$\begin{aligned} \lim _{t\rightarrow \infty }A_1=1,\quad \lim _{t\rightarrow \infty }\frac{A_2}{t}=c, \quad \lim _{t\rightarrow \infty }\frac{B_1}{t}=\frac{2}{\sqrt{3}}c, \quad \lim _{t\rightarrow \infty }\frac{B_2}{t}=c \end{aligned}$$

for a constant $c>0$. Hence, from the third term in $|F_a|^2$, we see $t^{-2}f^+$ is bounded as $t\rightarrow \infty$. Thus, from the first term, $g^+$ must be bounded. We also quickly see that if $f^+,g^+$ are both bounded then from the formulae for $|F_a|^2$ and $|\dot{a}|^2$ we see that $|F_A|^2$ is bounded as well. $\square$

We have a 1-parameter family of reducible invariant $G_2$-instantons on the BGGG $G_2$-manifold which have gauge group $U(1)\subseteq \mathrm{SU}(2)$: they are given in Corollary 1(b) with $x_2=x_3=0$ and have bounded (in fact, decaying) curvature. We start with our non-existence result, which shows that a 2-parameter family of initial conditions leads to local $G_2$-instantons which either do not extend with bounded curvature or can only extend as one of the above abelian instantons.

##### Theorem 8

Let *A* be a $\mathrm{SU}(2)^2\times U(1)$-invariant $G_2$-instanton with gauge group $\mathrm{SU}(2)$ defined in a neighbourhood of $\{0\}\times S^3$ on the BGGG $G_2$-manifold $\mathbb {R}^4\times S^3$ smoothly extending over $P_1$ as given by Proposition 7.

If $f_1^+\le \frac{1}{2}$, or $g_1^+\ge 0$ with $g_1^+\ge f_1^+$, then *A* extends globally to $\mathbb {R}^4\times S^3$ with bounded curvature if and only if *A* has gauge group *U*(1) and is given in Corollary 1(b) with $x_2=x_3=0$.

##### Proof

If $g^+\equiv 0$ then we obtain an abelian instanton as in Corollary 1(b) with $x_2=x_3=0$. Suppose, for a contradiction, that $g^+$ is not identically zero and that *A* is defined for all *t* has bounded curvature. By Lemma 6 and Remark 11 we may assume without loss of generality that $g_1^+>0$ and thus $g^+>0$ for all *t*.

Let $F=f^+/A_1$ and $G=g^+/A_1$ and let $s=r-\frac{9}{4} \in [0,\infty )$ be given as in (2.26). If we let $f'=\frac{df}{ds}$ then (4.14), (4.15) are equivalent to

4.22 $$\begin{aligned} F'=-G^2\quad \text {and}\quad G'=(H-F)G, \end{aligned}$$

where

4.23 $$\begin{aligned} H=\frac{1}{2}\left( \frac{2}{A_1^2}+\frac{1}{B_2^2} -\frac{A_2^2+B_1^2+B_2^2}{A_1A_2B_1B_2}\right) =1 -\frac{5\left( r-\frac{9}{20}\right) ^2-\frac{27}{10}}{r(r-3/4)(r+9/4)}. \end{aligned}$$

Notice that *H* takes values in (0, 1), is increasing, and $\lim _{s\rightarrow \infty }H(s)=1$.

Suppose first that $f_1^+\le \frac{1}{2}$. Since $f^+=f_1^+t+O(t^3)$ and $A_1=t/2+O(t^3)$ by Example 2, we see that $F(0)=2f_1^+\le 1$. Moreover, *F* is strictly decreasing by (4.22) as $G>0$, so there is $\epsilon >0$ so that $F(s)\le 1-\epsilon$ for all $s>0$. As $H(s)\rightarrow 1$, there exists $s_0>0$ so that $H(s)-F(s)>\frac{\epsilon }{2}$ for all $s\ge s_0$. We deduce from (4.22) that $G'>\frac{\epsilon }{2}G$ for all $s\ge s_0$ as $G>0$, and hence $G\ge e^{\epsilon s/2}$. Therefore, $g^+$ grows at least exponentially, so either the solution explodes for a finite *t*, or $F_A$ is unbounded by Lemma 7, giving a contradiction.

Now suppose $g_1^+\ge f_1^+$. Then $G(0)-F(0)\ge 0$ and one sees that $G(s)-F(s)>0$ and is increasing for small $s>0$ using $g^+=g_1^+t+u_2(0)t^3+O(t^5)$, $f^+=f_1^+t+u_1(0)t^3+O(t^5)$ and the formulae (4.16), (4.17), where the values $C_1(0),C_2(0)$ for the BGGG metric are given in Example 2.

We see from (4.22) that

4.24 $$\begin{aligned} (G-F)'=(H+G-F)G. \end{aligned}$$

Therefore, when $G-F=0$ we must have $(G-F)'=HG>0$. As $G-F$ is initially increasing, it therefore cannot have any zeros for $s>0$, which means that $G-F>0$ for all $s>0$. We deduce that $-F>-G$ and hence, by (4.22), $G'>(H-G)G$.

If $F(s)\le 1$ for some *s*, then we are in the same situation as the previous case of $f_1^+\le \frac{1}{2}$, which leads to a contradiction. If instead $F(s)>1$ for all *s* then *F* is bounded below, so as *F* is strictly decreasing we need from (4.22) that $\lim _{s\rightarrow \infty }G(s)=0$. Hence, as $H(s)\rightarrow 1$, there exists $s_0$ so that $G'>\frac{1}{2}G$ for all $s\ge s_0$, which implies that $g^+$ grows at least exponentially. This again gives a contradiction by Lemma 7. $\square$

##### Remark 12

The above proof of non-existence of irreducible instantons for $f_1^+\le \frac{1}{2}$ immediately extends to the Bogoyavlenskaya metrics by the asymptotics in (4.21). The proof for $g_1^+\ge 0$ and $g_1^+\ge f_1^+$ would also extend if we knew that *H* given in (4.23) continued to be positive for all $t>0$ for the Bogoyavlenskaya metrics.

We now give our existence result, which provides a full 2-parameter family of irreducible $\mathrm{SU}(2)^2\times U(1)$-invariant $G_2$-instantons with gauge group $\mathrm{SU}(2)$ on the BGGG $G_2$-manifold.

##### Theorem 9

Let *A* be a $\mathrm{SU}(2)^2\times U(1)$-invariant $G_2$-instanton with gauge group $\mathrm{SU}(2)$ defined in a neighbourhood of $\{0\}\times S^3$ on the BGGG $G_2$-manifold $\mathbb {R}^4\times S^3$ smoothly extending over $P_1$ as given by Proposition 7.

If $f_1^+\ge \frac{1}{2}+g_1^+>\frac{1}{2}$, then *A* extends globally to $\mathbb {R}^4\times S^3$ with bounded curvature.

##### Proof

Recall the notation from the proof of Theorem 8. The conditions in the statement are equivalent to $F(0)-1\ge G(0)>0$. Hence, by Lemma 6, we have that $G(s)>0$ for all *s*. Now observe from (4.22) that since the function *H* in (4.23) takes values in (0, 1) we have

4.25 $$\begin{aligned} \frac{d}{ds}\big ((F-1)^2-G^2\big )=2(1-H)G^2>0. \end{aligned}$$

As $(F-1)^2-G^2\ge 0$ at $s=0$, we have that $(F-1)^2-G^2>0$ for all $s>0$. Thus $(F-1)^2>G^2>0$ and since $F(0)>1$ this means that $F(s)>1$ for all *s*.

By (4.22), *F* is decreasing and thus *F* is bounded as it is bounded below (by 1). We also know that $0<G<F-1$ so *G* is also bounded. As *H* is also bounded, we deduce that a long time solution to the ODEs (4.22) must exist.

Since *F* is bounded below by 1, decreasing and exists for all *s* we must have again from (4.22) that $G(s)\rightarrow 0$ as $s\rightarrow \infty$, and that $\lim _{s\rightarrow \infty }F(s)$ exists and equals some constant greater or equal to 1. Hence, both $f^+$ and $g^+$ are bounded and so *A* has bounded curvature by Lemma 7. $\square$

##### Remark 13

Via the asymptotics in (4.21), we see that the function *H* in (4.23) for any given Bogoyavlenskaya metric is always bounded above by some $C\ge 1$ (possibly depending on the metric, though one might hope to show that $C=1$). Thus, the proof of Theorem 9 extends to prove the existence of $G_2$-instantons with bounded curvature for $f_1^+\ge \frac{C}{2}+g_1^+>0$ in these cases.

Given a $G_2$-instanton on the BGGG $\mathbb {R}^4\times S^3$ as in Theorem 9 we can evaluate its holonomy around the finite circle at infinity, which is a *U*(1) transformation. In particular, if we fix $g_1^+>0$, we obtain

4.26 $$\begin{aligned} Hol_{\infty } : \left( \tfrac{1}{2} + g_1^+ , + \infty \right) \rightarrow U(1) \subset \mathrm{SU}(2), \end{aligned}$$

which is the map that takes $f_1^+$ to this limit holonomy. It is natural to ask about the image of this map, which we now show is all of *U*(1).

##### Corollary 3

For any fixed $g_1^+ >0$, the map (4.26) is surjective.

##### Proof

From (4.25) we conclude that for all $s>0$

$$\begin{aligned}&(F(s)-1)^2> (F(s)-1)^2-G(s)^2 > (F(0)-1)^2-G(0)^2\\&\quad = \left( 2f_1^+ -1\right) ^2 -\left( 2 g_1^+\right) ^2. \end{aligned}$$

Since $F(s)>1$ for all *s* by the proof of Theorem 9, we deduce in fact that

$$\begin{aligned} F(s) > 1 + \sqrt{\left( 2f_1^+-1\right) ^2 -\left( 2 g_1^+\right) ^2 } \end{aligned}$$

for all $s>0$. Moreover, as *F* is decreasing by (4.22), we have that

4.27 $$\begin{aligned} F_{\infty } \left( f_1^+\right) := \lim _{ t \rightarrow + \infty } F(t) \in \left[ 1 + \sqrt{\left( 2f_1^+-1\right) ^2 -\left( 2 g_1^+\right) ^2 } \ , \ 2f_1^+ \right] . \end{aligned}$$

Hence, for any fixed $g_1^+$, we can vary $f_1^+ > \frac{1}{2}+g_1^+$ to ensure that $F_{\infty }$ is as large as we want. By continuous dependence with respect to initial conditions for ODEs, we have that $F_{\infty }$ varies continuously with $f_1^+$, and so the image of the map $F_{\infty } : ( \frac{1}{2} + g_1^+ , + \infty ) \rightarrow \mathbb {R}$ contains at least the interval $( 1+2g_1^+ , + \infty )$.

Now let $\gamma _t$ be the circle parametrized by

$$\begin{aligned} \gamma _t(\theta ) = \big (t , \exp _{(1,1)}(2 \pi \theta T_1^+) \big )\subseteq \mathbb {R}^+_t \times S^3 \times S^3 , \end{aligned}$$

for $\theta \in [0,1]$. Then, the holonomy of $A=a(t)$ around $\gamma _t$ is

$$\begin{aligned} Hol(\gamma _t)= & {} \exp \left( \int _{\gamma _t} a(t) \right) = \exp \left( \int _{\gamma _t} A_1(t)^2 F(t) T_1\otimes \eta _1^+ \right) \\= & {} \exp \big ( 2\pi A_1(t)^2 F(t) T_1\big ). \end{aligned}$$

Taking the limit as $t \rightarrow + \infty$ and recalling (4.21) gives $Hol_{\infty }= \exp (2 \pi F_{\infty }T_1)$. The surjectivity of $F_{\infty }$ onto $( 1+2g_1^+ , + \infty )$ proves the desired result. $\square$

##### Remark 14

The proofs of Theorem 9 and Corollary 3 show that for the $G_2$-instantons *A* constructed we have $F\rightarrow F_{\infty } \ge 1$ and $G\rightarrow 0$ at infinity. Moreover, if $F_{\infty }>1$ (which occurs if $f_1^+>\frac{1}{2}+g_1^+$) then (4.22) implies that *G* tends to 0 at an exponential rate. Observe that the abelian $G_2$-instantons of 1(b) with $x_2=x_3=0$ are given by $F=x_1\in \mathbb {R}$ and $G=0$. Hence, *A* is asymptotic to an abelian $G_2$-instanton, with exponential rate of convergence if $F_{\infty }>1$. Moreover, using Lemma 2 and (4.21) we may compute the pointwise norm of the curvature $F_A$ of *A* satisfies

$$\begin{aligned} \vert F_A \vert \sim 2\sqrt{\frac{A_1^4}{A_2^4}+\frac{A_1^4}{B_2^4} } =O(t^{-2}), \end{aligned}$$

which proves they have quadratically decaying curvature.

By contrast, in Proposition 5, we showed that the irreducible $\mathrm{SU}(2)^2\times U(1)$-invariant $G_2$-instantons for the BS metric are asymptotic to an irreducible connection and the rate of convergence is $O(t^{-3})$.

In summary, on the BGGG $G_2$-manifold $\mathbb {R}^4\times S^3$, we have shown non-existence for irreducible $\mathrm{SU}(2)^2\times U(1)$-invariant $G_2$-instantons with gauge group $\mathrm{SU}(2)$ and bounded curvature for $g_1^+>0$ and $f_1^+\le \frac{1}{2}$ or $g_1^+\ge f_1^+$, and existence for $f_1^+\ge \frac{1}{2}+g_1^+>\frac{1}{2}$. This currently leaves open the region where $0<f_1^+-\frac{1}{2}<g_1^+<f_1^+$. Some numerical investigation indicates that some of these initial conditions may lead to globally defined instantons with bounded curvature and some may not.

#### Solutions smoothly extending on $P_{{{\mathrm{id}}}}$

We now turn our attention to the more difficult case of solutions to the $\mathrm{SU}(2)^2\times U(1)$-invariant $G_2$-instanton equations on $\mathbb {R}^4\times S^3$ which smoothly extend on the bundle $P_{{{\mathrm{id}}}}$. Here the ODE system does not simplify, but we obtain a 1-parameter family of local solutions in a neighbourhood of the singular orbit. Although the strategy of proof remains the same as in our earlier similar results, the analysis is more involved. In order to ease computations, we use the Taylor expansion for a smooth $\mathrm{SU}(2)^2 \times U(1)$-symmetric $G_2$-holonomy metric in a neighbourhood of a singular orbit $\lbrace 0 \rbrace \times S^3$ at $t=0$, computed in (A.4)–(A.7), which depends on constants *b*, *c*.

##### Proposition 8

Let $X \subset \mathbb {R}^4\times S^3$ contain the singular orbit $\{0\}\times S^3$ of the $\mathrm{SU}(2)^2 \times U(1)$ action and be equipped with an $\mathrm{SU}(2)^2 \times U(1)$-invariant holonomy $G_2$-metric. There is a 1-parameter family of $\mathrm{SU}(2)^2 \times U(1)$-invariant $G_2$-instantons *A* with gauge group $\mathrm{SU}(2)$ in a neighbourhood of the singular orbit in *X* smoothly extending over $P_{{{\mathrm{id}}}}$.

Moreover, in the notation of Proposition 6 and (4.13), any such $G_2$-instanton *A* can be written as in (4.8) with $f^{\pm }$, $g^{\pm }$ solving the ODEs (4.9)–(4.12) subject to

$$\begin{aligned} f^{+}(t)&= \frac{2}{t} + \left( \frac{\left( b_0^-\right) ^2}{4}-\frac{1}{4b^2} -4c \right) t \\&\quad + \left( \frac{35 \left( b^2\left( b_0^-\right) ^2- \frac{16}{7} \right) b^2 \left( b_0^-\right) ^2 + 112 (b^2 c +12) b^2 c +22}{480 b^4} \right) t^3 + O(t^5 ),\\ g^+(t)&= \frac{2}{t} + \left( \frac{\left( b_0^-\right) ^2}{4} + 2c \right) t\\&\quad + \left( \frac{35 \left( b^2\left( b_0^-\right) ^2- \frac{16}{7} \right) b^2 \left( b_0^-\right) ^2 + 112 (b^2 c +12) b^2 c +22}{480 b^4} \right) t^3 + O(t^5 ), \\ f^-(t)&= b_0^- + \frac{b_0^-}{4b^2} \left( b^2 \left( b_0^-\right) ^2 -2\right) t^2 + O(t^4), \\ g^{-}(t)&= b_0^- + \frac{b_0^-}{4b^2} \left( b^2 \left( b_0^-\right) ^2 -2\right) t^2 + O(t^4), \end{aligned}$$

for $$b_0^- \in \mathbb {R}$$.

##### Proof

On $P_{{{\mathrm{id}}}}$, the singular initial value problem to be solved has

$$\begin{aligned} f^-(t)= & {} b_0^- + t^2 v_1(t), \quad g^{-}(t)=b_0^- + t^2 v_2(t),\\ f^{+}(t)= & {} \frac{2}{t} + \left( b_2^+-4C_1(0) \right) t + t^3 u_1(t), \quad g^+(t)= \frac{2}{t} + \left( b_2^+-4C_2(0)\right) t + t^3 u_2(t), \end{aligned}$$

for some real analytic $v_1(t),v_2(t),u_1(t),u_2(t)$ by Lemma 5. Moreover, notice that from (A.4)–(A.7) we have $C_1(0)=c$ and $C_2(0)=-\frac{1+8 cb^2}{16 b^2}$ and in the following we will write the coefficients of the metric in terms of $b,c \in \mathbb {R}$. The ODEs in Proposition 6 then turn into the following ones for $X(t)=(u_1(t),u_2(t),v_1(t),v_2(t))$:

$$\begin{aligned} \frac{dX}{dt} = \frac{M_{-3}\left( b_0^- , b_2^+\right) }{t^3}+ \frac{M_{-1}(X(t))}{t} + f(t,X(t)), \end{aligned}$$

where *f*(*t*, *X*(*t*)) is real analytic in both entries and

$$\begin{aligned} M_{-3}\left( b_0^- , b_2^+\right) = \left( \left( b_0^-\right) ^2-4b_2^+- \frac{1}{b^2}, \left( b_0^-\right) ^2-4b_2^+- \frac{1}{b^2}, 0 , 0 \right) . \end{aligned}$$

For this to have a real analytic solution *X*(*t*) we must have $M_{-3}=0$ which requires that $4b_2^+=(b_0^-)^2-\frac{1}{b^2}$. In that case we have

$$\begin{aligned} M_{-1}(X(0))&= \Big ( -6u_1(0)+ 2b_0^- v_2(0) , -3 u_1(0)-3u_2(0)+ b_0^- v_1(0) + b_0^- v_2(0), \\&\quad -6v_1(0)+4v_2(0), 2v_1(0)-4v_2(0) \Big ) + K(b_0^-), \end{aligned}$$

where $K(b_0^-)\in \mathbb {R}^4$ is a constant only depending on $b_0^-$ and the metric. For a real analytic solution to exist we need $M_{-1}(X(0))=0$. As this is a linear equation and $dM_{-1}(X(0))$ is always an isomorphism, it can be uniquely solved for any $K(b_0^-)$. The unique solution of $M_{-1}(X(0))=0$ can be written as

4.28 $$\begin{aligned} u_1(0)= & {} u_2(0)= \frac{35 \left( b^2\left( b_0^-\right) ^2- \frac{16}{7} \right) b^2 \left( b_0^-\right) ^2 + 112 \left( b^2 c +12\right) b^2 c +22}{480 b^4},\nonumber \\ v_1(0)= & {} v_2(0) = \frac{b_0^-}{4b^2} \left( b^2 \left( b_0^-\right) ^2 -2\right) . \end{aligned}$$

We now use the existence and uniqueness theorem for initial value problems of [18]. This guarantees that for each $b_0^- \in \mathbb {R}$ there is a unique solution to the system

$$\begin{aligned} \frac{dX}{dt} = \frac{M_{-1}(X(t))}{t} + f(t,X(t)), \end{aligned}$$

provided that $M_{-1}(X(0))=0$ and $d M_{-1}(X)$ has no eigenvalues in the positive integers. We showed above that we can always find a unique *X*(0) such that $M_{-1}(X(0))=0$. Moreover, the eigenvalues of $dM_{-1}$ can be computed to be $-8,-6,-3,-2$. Hence, for each $b_0^-\in \mathbb {R}$ there is indeed a unique solution *X*(*t*) to the system above. This yields a unique $G_2$-instanton as in the statement determined by $b_0^-$. $\square$

##### Remark 15

Since the BS, BGGG and Bogoyavlenskaya $G_2$-metrics all have SU(2)$^2 \times U(1)$-symmetry, Proposition 8 yields $G_2$-instantons in these cases. In particular, in the BS case we have $c=-\frac{1}{24 b^2}$, $b=\frac{1}{\sqrt{3}}$ and these $G_2$-instantons coincide with those given in Proposition 4.

In light of the existence result in Theorem 5 and the local existence result in Proposition 8, it is certainly an interesting non-trivial question which members of the 1-parameter family of local $G_2$-instantons from Proposition 8 extend on $P_{{{\mathrm{id}}}}$ on the BS, BGGG or Bogoyavlenskaya $\mathbb {R}^4\times S^3$.

Another natural problem for further study is to understand the limits of the family of instantons constructed in Theorem 9, and their possible relationship to any extensions of the local instantons given in Proposition 8. We saw in Proposition 5 that global $G_2$-instantons on the BS $\mathbb {R}^4\times S^3$ have a limit at infinity given by a canonical connection on the link $S^3\times S^3$ of the asymptotic cone. For the instantons constructed in Theorem 9 we know, by Remark 14, that these are asymptotic to the abelian $G_2$-instantons with a rate depending on the asymptotic connection. It is also certainly an interesting problem to investigate the behaviour of the family of instantons from Theorem 9 when one or both of $f_1^+$ and $g_1^+$ go to infinity. We would expect bubbling phenomena as in the BS case in Theorem 6, with possible relationship to the ASD instantons on Taub–NUT found in [11]. The lack of an explicit formula for our instantons makes the bubbling analysis more difficult.

One other interesting problem is to investigate the behaviour of $G_2$-instantons as the underlying metric is deformed. For instance, Remark 13 shows how to adapt the proof of existence in Theorem 9 to the Bogoyavlenskaya $G_2$-manifolds, and we would want to analyse these instantons as the size of the circle at infinity gets very large or small. When it gets very large we expect them to resemble $G_2$-instantons for the BS metric given in Theorem 7. When it gets very small, there may be a relation with Calabi–Yau monopoles on the deformed conifold (as in [21]).

## Appendix A: SU(2)$^2$-invariant tensors

In this appendix, we use Eschenburg–Wang’s technique [12] to determine when a metric or connection extends smoothly over a singular orbit $Q=\mathrm{SU}(2)\times \mathrm{SU}(2)/ \Delta \mathrm{SU}(2)\cong S^3$ in $X=\mathbb {R}^4 \times S^3$. The relevant group diagram is $I(\mathrm{SU}(2)\times \mathrm{SU}(2); \lbrace 1 \rbrace ; \mathrm{SU}(2))$ and so the principal orbits are topologically $S^3 \times S^3$. We will often identify $\mathrm{SU}(2)$ with the unit quaternions.

The normal bundle *NQ* to *Q* is $\mathbb {R}^4 \times S^3$ and is homogeneously constructed by $NQ= (\mathrm{SU}(2) \times \mathrm{SU}(2) )\times _{ \mathrm{SU}(2)} \mathbb {H}$, where $\mathrm{SU}(2)$ acts on $\mathrm{SU}(2)\times \mathrm{SU}(2)$ diagonally and on $\mathbb {H}$ by left multiplication. Similarly, $TQ=(\mathrm{SU}(2)\times \mathrm{SU}(2) ) \times _{\mathrm{SU}(2)} {{\mathrm{im}}}(\mathbb {H})$, where $q\in \mathrm{SU}(2)$ acts on $x \in {{\mathrm{im}}}(\mathbb {H})$ by $q \cdot x = qx\overline{q}$. We also note that

$$\begin{aligned} T(NQ) \cong NQ\oplus \pi ^* TQ, \end{aligned}$$

where $\pi : NQ \rightarrow Q$ is the projection.

### A.1: Metrics

By the previous discussion, *T*(*NQ*) is modelled on $W=\mathbb {H}\oplus {{\mathrm{im}}}(\mathbb {H})$, with $a\in \mathrm{SU}(2)$ acting by $a \cdot (p,q)= (ap,a q \overline{a})$, for $(p,q) \in W$. Following [12], to determine which metrics extend smoothly over *Q* we seek a basis of $S^2(W)$ corresponding to the evaluation at $1 \in \mathbb {H}$ of homogeneous $\mathrm{SU}(2)$-equivariant polynomials $\mathbb {H} \rightarrow S^2(W)$ of minimal degree.

The equivariance condition implies that any such polynomial is of the form$x \mapsto \phi (x) \in S^2(W) \subset W \otimes W \cong {{\mathrm{End}}}(W)$, such that for $(p,q) \in W = \mathbb {H}\oplus {{\mathrm{im}}}(\mathbb {H})$ and $x\in \mathrm{SU}(2)$ we have

A.1 $$\begin{aligned} \phi (x) (p,q)&=\big (\phi _1(x)(p,q),\phi _2(x)(p,q)\big )\nonumber \\&=\big (x \phi _1 (1) ( \overline{x} p ,\overline{x} q x ), x \big (\phi _2(1) ( \overline{x} p,\overline{x} q x ) \big )\overline{x} \big ). \end{aligned}$$

- First we look at maps $x \mapsto \psi (x)(\cdot ) \in {{\mathrm{End}}}({{\mathrm{im}}}\mathbb {H})$ such that $\psi (x)(q)= x ( \psi (1) ( \overline{x} q x ) ) \overline{x}$ for $x\in \mathrm{SU}(2)$. The identity map is constant and so homogeneous of degree 0. We also have the homogeneous degree 4 polynomials $\psi (x)(q)=-xl\overline{x}qxl\overline{x}$ for $l\in \{i,j,k\}$. Given the coordinates $q=q_1i+q_2j+q_3 k \in {{\mathrm{im}}}( \mathbb {H})$ we have a canonical identification ${{\mathrm{End}}}({{\mathrm{im}}}(\mathbb {H}) \cong {{\mathrm{im}}}(\mathbb {H})^* \otimes {{\mathrm{im}}}(\mathbb {H})^*$. Using this identification, we have that the identity and degree 4 polynomials given, when evaluated at $x=1$, correspond to
  $$\begin{aligned} dq_1&\otimes&dq_1 + dq_2 \otimes dq_2 + dq_3 \otimes dq_3,\\ dq_1&\otimes&dq_1 - dq_2 \otimes dq_2 - dq_3 \otimes dq_3, \\ -dq_1&\otimes&dq_1 + dq_2 \otimes dq_2 - dq_3 \otimes dq_3,\\ -dq_1&\otimes&dq_1 - dq_2 \otimes dq_2 + dq_3 \otimes dq_3. \end{aligned}$$
- Now we consider maps $x \mapsto \psi (x)(\cdot ) \in {{\mathrm{End}}}( \mathbb {H})$ such that $\psi (x)(p)= x \psi (1)( \overline{x} p )$ for $x\in \mathrm{SU}(2)$. Fixing coordinates $p=p_0 + i p_1 + j p_2 + kp_3 \in \mathbb {H}$, we may identify ${{\mathrm{End}}}(\mathbb {H})$ with $\mathbb {H}^* \otimes \mathbb {H}^*$. Certainly, the constant maps given by the identity and the complex structures are $\mathrm{SU}(2)$-equivariant. The constant map corresponds to
  $$\begin{aligned} dp_0 \otimes dp_0 + dp_1 \otimes dp_1 + dp_2 \otimes dp_2 + dp_3 \otimes dp_3, \end{aligned}$$
  while the complex structures correspond to antisymmetric (anti-self-dual) 2-tensors. We also have homogeneous degree 2 polynomials, where $\psi (x)(p)=\langle p,xl\rangle xl$ for $l\in \{i,j,k\}$. These are $\mathrm{SU}(2)$-equivariant and correspond under evaluation at $x=1$ to
  $$\begin{aligned} dp_1 \otimes dp_1, \ dp_2 \otimes dp_2, \ dp_3 \otimes dp_3. \end{aligned}$$
- Finally, it suffices to consider maps $x\mapsto \phi (x)$ as in (A.1) with $\phi _1(x)(p,q)=\phi _1(x)(q)$ and $\phi _2(x)(p,q)=\phi _2(x)(p)$. We have the $\mathrm{SU}(2)$-equivariant linear polynomial $\phi (x)(p,q)=(qx,\frac{1}{2}(p\bar{x}-x\bar{p}))$, which in the coordinates as above corresponds at $x=1$ to
  $$\begin{aligned} \sum _{i=1}^3 dq_i\otimes dp_i+dp_i\otimes dq_i. \end{aligned}$$
  The equivariant homogeneous degree 3 polynomials
  $$\begin{aligned} \phi (x)(p,q)=(\langle q,xl_1\overline{x}\rangle xl_2,\langle p,xl_2\rangle xl_1\overline{x}), \end{aligned}$$
  for $l_1,l_2\in \{i,j,k\}$, then correspond under evaluation at $x=1$ to $dp_i\otimes dq_j+dq_i\otimes dp_j$ for $i,j\in \{1,2,3\}$.

#### Remark 16

As an alternative to the degree 4 polynomials we wrote down in the first bullet above, we could have used $\psi (x)(q)=\langle q, xl_1\overline{x} \rangle xl_2\overline{x}$, where $l_1,l_2 \in \lbrace i,j,k \rbrace$.

We now have enough information to analyze metrics of the form

A.2 $$\begin{aligned} g= dt^2 + \sum _{i=1}^3 (2A_i(t))^2 \eta _{i}^+ \otimes \eta _i^+ + (2B_i(t))^2 \eta _i^- \otimes \eta _i^-, \end{aligned}$$

where $\eta _i^{\pm }$ define bases for the diagonal and anti-diagonal copies of $\mathfrak {su}(2)$ in $\mathfrak {su}(2)\oplus \mathfrak {su}(2)$ as in Sect. 2.2. We embed $\mathbb {R}^4 \times S^3 \hookrightarrow \mathbb {H} \times \mathbb {H}$ and let $\mathrm{SU}(2) \times \mathrm{SU}(2)$ act via $(a_1,a_2) \cdot (p,q) = (a_1p,a_1 q \overline{a}_2)$. Using this action and the coordinates $p=p_0+ip_1+jp_2+kp_3$ and $q=q_0+iq_1+jq_2+kq_3$, we compute that, at $(t,1)\in \mathbb {R}^4\times S^3$ for $t\in \mathbb {R}$, the dual frames $\{T_i^{\pm }\}$ to the coframes $\{\eta _i^{\pm }\}$ satisfy

$$\begin{aligned} T_i^+ = t \frac{\partial }{\partial p_i}, \quad T_i^-=t \frac{\partial }{\partial p_i} + 2 \frac{\partial }{\partial q_i}, \quad \frac{\partial }{\partial t} = \frac{\partial }{\partial p_0}, \end{aligned}$$

for $i=1,2,3$. At $t=0$ the isotropy is $\Delta \mathrm{SU}(2)$ and the orbit *Q* is an $S^3$ whose tangent space at (0, 1) is $0\oplus {{\mathrm{im}}}(\mathbb {H})$. For $t\ne 0$ we have

A.3 $$\begin{aligned} \eta _i^+=\frac{1}{t}dp_i - \frac{1}{2}dq_i ,\quad \eta _i^- = \frac{1}{2}dq_i,\quad dt = dp_0. \end{aligned}$$

It is now easy to rewrite the metric in (A.2) in terms of the equivariant symmetric 2-tensors we found above. This gives

$$\begin{aligned} g&= dp_0^2 + \sum _{i=1}^3 \left( \frac{2A_i}{t} \right) ^2 dp_i \otimes dp_i - \sum _{i=1}^3 \frac{2A_i^2}{t} \left( dp_i \otimes dq_i + dq_i \otimes dp_i \right) \\&\quad + \sum _{i=1}^3 \left( A_i^2 + B_i^2 \right) dq_i \otimes dq_i \\&= \sum _{i=1}^4 dp_i^2 + C \sum _{i=1}^3 dq_i^2 + \sum _{i=1}^3 \bigg (\left( \frac{2A_i}{t} \right) ^2 -1 \bigg ) dp_i^2 + \sum _{i=1}^3 \left( A_i^2 + B_i^2- C \right) dq_i^2\\&\quad + D\sum _{i=1}^3 \left( dp_i \otimes dq_i + dq_i \otimes dp_i \right) -\sum _{i=1}^3\left( \frac{2A_i^2}{t}+D\right) \left( dp_i \otimes dq_i + dq_i \otimes dp_i \right) \end{aligned}$$

where *C* is some smooth even function of *t* and *D* is some smooth odd function of *t*. Eschenburg–Wang’s technique guarantees that *g* smoothly extends over *Q* if and only if, for $i=1,2,3$, $\left( 2A_i/t \right) ^2 -1$ is even and $O(t^2)$, $B_i^2+A_i^2-C$ is even and $O(t^4)$, and $\frac{2A_i^2}{t}+D$ is odd and $O(t^3)$. In other words, $A_i(t) = t/2 + O(t^3)$ and $B_i^2(t)=C(t) - t^2/4 + O(t^4)$; in particular notice that up to order $O(t^2)$ the $A_i$ and $B_i$ do not depend on $i=1,2,3$. Moreover, for *g* to extend to a metric we also require it to be positive definite. This implies that $A_i$, $B_i$ are sign definite for $t>0$ and $A_i(0)=0$, while $B_i(0) \ne 0$. We summarise these conclusions.

#### Lemma 8

The metric *g* in (A.2) extends smoothly (as a metric) over the singular orbit $Q=\mathrm{SU}(2)^2/ \Delta \mathrm{SU}(2)$ if and only if $A_i$, $B_i$ are sign definite for $t>0$ and:

- the $A_i$’s are odd with $\dot{A}_i(0)=1/2$;
- the $B_i$’s are even with $B_1(0)=B_2(0)=B_3(0) \ne 0$ and $\ddot{B}_1(0)=\ddot{B}_2(0)=\ddot{B}_3(0)$.

#### Remark 17

In fact, for our applications there is no restriction in having the metrics above being real analytic instead of smooth. As $G_2$ manifolds are Ricci-flat, the metric is real analytic in harmonic coordinates. The function *t* can be interpreted as the arclength parameter along a geodesic intersecting the principal orbits orthogonally, so it is a real analytic function of the harmonic coordinates, and thus the metric coefficients must be real analytic functions of *t*.

Using Lemma 8 and Eqs. (2.16)–(2.19) we can compute the first order terms in the Taylor expansion for a metric with holonomy $G_2$ in a neighbourhood of a singular orbit *Q* at $t=0$ to be

A.4 $$\begin{aligned} A_1(t)&= \frac{t}{2} + c t^3 + {\frac{ 96(22c{b}^{2}+1) cb^2+11}{640 b^{4}}} t^5 + \cdots \end{aligned}$$

A.5 $$\begin{aligned} A_2(t)&= \frac{t}{2} - \frac{1+ 8 c b^2}{16 b^2} t^3 - {\frac{ 11- 24( 32c{b}^{2}+1) cb^2}{640 b^{4}}} t^5 + \cdots \end{aligned}$$

A.6 $$\begin{aligned} B_1(t)&= b + \frac{1}{4 b} t^2 - \frac{7+8cb^2}{160 b^3} t^4 + \cdots \end{aligned}$$

A.7 $$\begin{aligned} B_2(t)&= b + \frac{1}{4 b} t^2 - \frac{13-8cb^2}{320 b^3} t^4 + \cdots \end{aligned}$$

We now confirm that the BS and BGGG metrics from Sect. 2.2 satisfy the conditions of Lemma 8. We use these formulae on a number of occasions.

#### Example 1

The BS metric on $\mathbb {R}^4\times S^3$ from Sect. 2.2.1 has $A_1=A_2=A_3$ and $B_1=B_2=B_3$ in (A.2), with expansions

$$\begin{aligned} A_1(t) = \frac{t}{2}-\frac{1}{8} t^3 +O(t^5), \quad B_1(t) = \frac{1}{\sqrt{3}} + \frac{\sqrt{3}}{4} t^2 - \frac{\sqrt{3}}{8} t^4 +O(t^6). \end{aligned}$$

#### Example 2

The BGGG metric on $\mathbb {R}^4\times S^3$ from Sect. 2.2.2 has $A_2=A_3$ and $B_2=B_3$ in (A.2), with expansions

$$\begin{aligned} A_1(t)= & {} \frac{t}{2}-\frac{7}{108} t^3 +O(t^5), \quad A_2(t) = \frac{t}{2}+\frac{1}{216} t^3 +O(t^5), \\ B_1(t)= & {} \frac{3}{2} + \frac{1}{6} t^2 - \frac{7}{648} t^4 + O(t^6), \quad B_2(t) = \frac{3}{2} + \frac{1}{6} t^2 - \frac{17}{1296} t^4 + O(t^6) . \end{aligned}$$

### A.2: Lie algebra-valued 1-forms

Let *G* be a compact Lie group with Lie algebra $\mathfrak {g}$. We now analyze the conditions to extend $\mathfrak {g}$-valued 1-forms of the form

A.8 $$\begin{aligned} b = \sum _{i=1}^3 b_i^+ \otimes \eta _i^+ + \sum _{i=1}^3 b_i^- \otimes \eta _i^- \end{aligned}$$

over the singular orbit *Q*. This a priori depends on how the (trivial) bundle $P= (\mathrm{SU}(2) \times \mathrm{SU}(2) ) \times G$ extends over *Q*. Such extensions are parametrized by (conjugacy classes) of isotropy homomorphisms $\mu : \mathrm{SU}(2) \rightarrow G$. Given $\mu$, we pull $P_{\mu }=\mathrm{SU}(2)^2 \times _{(\mathrm{SU}(2),\mu )} \mathfrak {g}$ back to $\mathbb {R}^4 \times S^3$, which determines the extension.

Then $\mathrm{SU}(2)$ acts on $\mathfrak {g}$ via ${{\mathrm{Ad}}}\circ \mu$ and we need a basis for ${{\mathrm{Hom}}}(W , \mathfrak {g})$ given by evaluation at 1 of homogeneous $\mathrm{SU}(2)$-equivariant polynomials $\mathbb {H} \rightarrow {{\mathrm{Hom}}}(W , \mathfrak {g})$, where $W = \mathbb {H}\oplus {{\mathrm{im}}}(\mathbb {H})$. Following [12], we seek homogeneous polynomials $x \mapsto \phi (x)$ such that for $(p,q) \in W$ we have, for $x\in \mathrm{SU}(2)$,

$$\begin{aligned} \phi (x) (p,q)= {{\mathrm{Ad}}}\circ \mu \left( x \right) \phi (1) \left( \overline{x}p, \overline{x} q x \right) . \end{aligned}$$

#### A.2.1: $G=U(1)$

Here, $\mu :\mathrm{SU}(2) \rightarrow U(1)$ must be trivial and $\mathfrak {g}=\mathbb {R}$. We also have ${{\mathrm{Hom}}}(W, \mathbb {R})\cong \mathbb {H}^*\oplus {{\mathrm{im}}}(\mathbb {H})^*$ and we are left with analyzing when a 1-form extends over *Q*. We describe $\mathrm{SU}(2)$-equivariant homogeneous polynomials in $\mathbb {H}$ with values in ${{\mathrm{im}}}(\mathbb {H})^*$ and $\mathbb {H}^*$ independently.

- First we look for homogeneous polynomials $\mathbb {H} \rightarrow {{\mathrm{im}}}(\mathbb {H})^*$, given by $x\mapsto \psi (x)$ such that $\psi (x)(q)=\psi (1)(\overline{x}qx)$ for $x\in \mathrm{SU}(2)$. These are generated by the degree 2 polynomials $\psi (x)(q)=\langle qx, xl \rangle$, where $l \in \lbrace i,j,k \rbrace$. Under evaluation at $x=1$ these correspond to the 1-forms $dq_i$ for $i=1,2,3$.
- Next we look for homogeneous polynomials $\mathbb {H} \rightarrow \mathbb {H}^*$ given by $x \mapsto \psi (x)$ such that $\psi (x)(p)=\psi (1)(\overline{x} p )$ for $x\in \mathrm{SU}(2)$. These are generated by the degree 1 polynomials $\psi (x)(p)= \langle p, x l \rangle$, where $l \in \lbrace 1,i,j,k \rbrace$, which correspond under evaluation at $x=1$ to the $dp_i$’s for $i=0,1,2,3$.

We now consider extending the 1-form

A.9 $$\begin{aligned} b = \sum _{i=1}^3 b_i^+ \eta _i^+ + \sum _{i=1}^3 b_i^- \eta _i^- = \sum _{i=1}^3 \frac{b_i^+}{t} dp_i + \sum _{i=1}^3 \frac{b_i^- - b_i^+}{2} dq_i, \end{aligned}$$

where we used (A.3). Using the homogeneous polynomials in $\mathbb {H}$ computed above and Eschenburg–Wang’s technique, we immediately deduce the following.

##### Lemma 9

The 1-form *b* as given in (A.9) extends over the singular orbit$Q=\mathrm{SU}(2)^2/\Delta \mathrm{SU}(2)$ if and only if the $b_{i}^{\pm }$’s are even and $b_i^{\pm }(0)=0$ for $i=1,2,3$.

#### A.2.2: $G=\mathrm{SU}(2)$

In this case, using our earlier notation, $\mu :\mathrm{SU}(2) \rightarrow \mathrm{SU}(2)$ must be either the identity $\mu ={{\mathrm{id}}}$ (up to conjugacy), or the trivial homomorphism $\mu =1$. Then, $\mathfrak {g}=\mathfrak {su}(2)\cong {{\mathrm{im}}}(\mathbb {H})$ and ${{\mathrm{Ad}}}\circ \mu$ is either the adjoint action ${{\mathrm{Ad}}}$ or trivial, respectively. We shall denote the respective bundles by $P_{{{\mathrm{id}}}}=(\mathrm{SU}(2) \times \mathrm{SU}(2))\times _{(\Delta \mathrm{SU}(2), {{\mathrm{id}}})} \mathrm{SU}(2)$ and $P_{1}=(\mathrm{SU}(2) \times \mathrm{SU}(2))\times _{(\Delta \mathrm{SU}(2), 1)} \mathrm{SU}(2)$. The main result of this section considers the problem of extending $\mathfrak {su}(2)$-valued 1-forms as in (A.8).

##### Lemma 10

Let *b* be an $\mathfrak {su}(2)$-valued 1-form as in (A.8). Write $b_i^{\pm }= \sum _{j=1}^3 b_{ij}^{\pm } T_j$, where $\lbrace T_i \rbrace _{i=1}^3$ is a standard basis for $\mathfrak {su}(2)$. Then the 1-form *b* extends over the singular orbit $Q=\mathrm{SU}(2)^2/\Delta \mathrm{SU}(2)$ on the bundle $P_{\mu }$ if:

- $\mu ={{\mathrm{id}}}$ and for $i=1,2,3$, $b_{ii}^{\pm }$ are even and there are $c_0^{-}, c_2^{\pm } \in \mathbb {R}$ such that
  $$\begin{aligned} b_{ii}^+ = c_2^+ t^2 + O(t^4), \quad b^-_{ii}= c_0^- + c_2^- t^2 + O(t^4); \end{aligned}$$
  and for $i \ne j$, $b_{ij}^{\pm }=O(t^4)$ are even;
- $\mu =1$ and the $b_{ij}^{\pm }$’s are even with $b_{ij}^{\pm }(0)=0$.

The rest of this Appendix is concerned with the proof of Lemma 10.

#### Case $\mu ={{\mathrm{id}}}$

Here, we may write ${{\mathrm{Ad}}}(x) q= x q \overline{x}$ and ${{\mathrm{Hom}}}(W, {{\mathrm{im}}}(\mathbb {H})) \cong ( {{\mathrm{im}}}(\mathbb {H})\otimes \mathbb {H}^* )\oplus ( {{\mathrm{im}}}(H)\otimes {{\mathrm{im}}}(\mathbb {H})^* )$. As before, we shall analyze $\mathrm{SU}(2)$-equivariant homogeneous polynomials in $\mathbb {H}$ with values in each of the components independently.

- We begin by looking for homogeneous polynomials $\mathbb {H} \rightarrow {{\mathrm{im}}}(\mathbb {H})\otimes \mathbb {H}^*$ given by $x \mapsto \psi (x)$ such that $\psi (x)(q)=x (\psi (1)(\overline{x} q x )) \overline{x}$ for $x\in \mathrm{SU}(2)$. We have the constant polynomial corresponding to the identity, which is
  $$\begin{aligned} T_1\otimes dq_1 + T_2\otimes dq_2 + T_3\otimes dq_3 . \end{aligned}$$
  We also see that the degree 4 polynomials $\psi (x)(q)=\langle q, xl_1\overline{x} \rangle xl_2\overline{x}$, where $l_1,l_2\in \lbrace i,j,k \rbrace$, generate the space of $T_j \otimes dq_i$ for $i,j=1,2,3$ when evaluated at $x=1$.
- Next we look for homogeneous polynomials $\mathbb {H} \rightarrow {{\mathrm{im}}}(\mathbb {H})\otimes \mathbb {H}^*$ given by $x \mapsto \psi (x)$ such that $\psi (x)(p)=x (\psi (1)(\overline{x} p )) \overline{x}$ for $x\in \mathrm{SU}(2)$. The degree 1 polynomials $\psi (x)(p)= pl \overline{x} + \langle p, x l \rangle$, where $l \in \lbrace 1,i,j,k \rbrace$, correspond under evaluation at $x=1$ to the maps
  $$\begin{aligned} T_1&\otimes dp_1 + T_2 \otimes dp_2 + T_3 \otimes dp_3 , \quad T_1 \otimes dp_0 - T_3 \otimes dp_2 + T_2 \otimes dp_3 , \\ T_2&\otimes dp_0 + T_3 \otimes dp_1 - T_1 \otimes dp_3, \quad T_3 \otimes dp_0 - T_2 \otimes dp_1 + T_1 \otimes dp_2. \end{aligned}$$
  We also have $\mathrm{SU}(2)$-equivariant maps $\psi (x)(q)=\langle xl_1 , p \rangle xl_2\overline{x}$, for $l_1\in \{1,i,j,k\}$, $l_2\in \{i,j,k\}$ which are homogeneous of degree 3. Taking $x=1$, these generate $T_j\otimes dp_i$, for $i=0,1,2,3$ and $j=1,2,3$.

Recall that our goal is to consider the problem of extending the $\mathfrak {su}(2)$-valued 1-form

$$\begin{aligned} b = \sum _{i=1}^3 b_i^+ \otimes \eta _i^+ + \sum _{i=1}^3 b_i^- \otimes \eta _i^- = \sum _{i=1}^3 \frac{b_i^+}{t} \otimes dp_i + \sum _{i=1}^3 \frac{b_i^--b_i^+}{2} \otimes dq_i, \end{aligned}$$

where we used (A.3). Since $b_i^{\pm }\in \mathfrak {su}(2)$, we can write $b_i^{\pm }= \sum _{j=1}^3 b_{ij}^{\pm } T_j$ and

$$\begin{aligned} b =&\frac{b_{11}^+}{t} \sum _{i=1}^3 T_i \otimes dp_i + \sum _{i=1}^3 \frac{b^+_{ii}- b_{11}^+}{t} T_i \otimes dp_i + \sum _{i \ne j} \frac{b^+_{ij}}{t} T_i \otimes dp_j \\&+ \frac{b_{11}^-}{2} \sum _{i=1}^3 T_i \otimes dq_i + \sum _{i=1}^3 \frac{b_{ii}^- - b_{ii}^+ - b_{11}^-}{2} T_i \otimes dq_i + \sum _{i \ne j} \frac{b_{ij}^- - b_{ij}^+}{2} T_i \otimes dq_j. \end{aligned}$$

Given the homogeneous polynomials in $\mathbb {H}$ computed above, we conclude that *b* extends smoothly over *Q* on $P_{{{\mathrm{id}}}}$ if and only if: $\frac{b_{11}^+}{t}$ is odd; $\frac{b_{11}^-}{2}$ is even; $\frac{b^+_{ii}- b_{11}^+}{t}=O(t^3)$ and, for $i \ne j$, $\frac{b^+_{ij}}{t}=O(t^3)$ are odd; $\frac{b_{ii}^- - b_{ii}^+ - b_{11}^-}{2}=O(t^4)$ and, for $i \ne j$, $\frac{b_{ij}^- - b_{ij}^+}{2}=O(t^4)$ are even. Hence, the $b_{ij}^+$ are all even and $b_{11}^+ = O(t^2)$, $b_{ii}^+ = b_{11}^+ +O(t^4)$ (so the $O(t^2)$ terms in all $b_{ii}^+$ coincide) and for $i \ne j$ we have $b_{ij}^+=O(t^4)$. Thus, the $b_{ij}^-$ must all be even, $b_{ii}^-=b_{11}^- + b_{ii}^+ + O(t^4)$ (so, up to order $O(t^4)$ the $b_{ii}^-$ do not depend on *i*) and for $i \ne j$ we have $b_{ij}^- = b_{ij}^+ +O(t^4)$. This proves the first part of Lemma 10.

#### Case $\mu =1$

Here, ${{\mathrm{Ad}}}\circ \mu (x) q= q$, so we require homogeneous $\mathrm{SU}(2)$-equivariant polynomials $\mathbb {H}\rightarrow {{\mathrm{Hom}}}(W,\mathbb {R}^3)$ where the action of $\mathrm{SU}(2)$ on $\mathbb {R}^3$ is trivial. This is essentially the same as the situation where the gauge group $G=U(1)$. Therefore, as in that setting, we have degree 2 polynomials corresponding to $T_j\otimes dq_i$ for $i,j=1,2,3$ and degree 1 polynomials corresponding to $T_j\otimes dp_i$ for $i=0,1,2,3$ and $j=1,2,3$.

We can now consider the problem here of extending the $\mathfrak {su}(2)$-valued 1-form *b* in (A.8) over *Q*. As before, we write $b_i^{\pm }= \sum _{j=1}^3 b_{ij}^{\pm } T_j$ and deduce from (A.3) that

$$\begin{aligned} b&= \sum _{i,j=1}^3 \left( \frac{b_{ij}^+}{t} T_j \otimes dp_i + \frac{b_{ij}^- - b_{ij}^+}{2} T_j \otimes dq_i \right) . \end{aligned}$$

Hence *b* extends smoothly over *Q* on $P_1$ if and only if: $\frac{b_{ij}^+}{t}$ are odd, while the $\frac{b_{ij}^- -b_{ij}^+}{2}$ are even and must vanish at $t=0$. In other words, for all $i,j \in \lbrace 1,2,3 \rbrace$, $b_{ij}^{\pm } = O(t^2)$ and is even. This completes the proof of Lemma 10.

## Footnotes

1 For further background on $G_2$-manifolds, the reader may wish to consult Joyce’s book [16].

2 An SU(3)-structure on an almost complex 6-manifold (*M*, *J*) can be given by a pair of a real (1, 1)-form $\omega$ and a real 3-form $\Omega _2$ such that

$$\begin{aligned} \omega \wedge \Omega _2 =0 , \quad \omega ^3 = - \frac{8}{3} \Omega _1 \wedge \Omega _2 , \end{aligned}$$

where $$\Omega _1=-J\Omega _2$$.

3 The nomenclature “Hitchin flow” is somewhat misleading since the system (2.2) is not parabolic in any usual sense and it does not satisfy the typical regularity properties of geometric flows [4].

4 There are, in fact, distinct $\mathrm{SU}(2)^3$-invariant torsion-free $G_2$-structures on $\mathbb {R}^4\times S^3$ inducing the same asymptotically conical Bryant–Salamon metric, determined by their image in $H^3(S^3\times S^3)$.

5 We thank Lorenzo Foscolo and Mark Haskins for bringing the metrics in [3] to our attention.
